# Overview obstacle maps for obstacle‐aware navigation of autonomous drones

**DOI:** 10.1002/rob.21863

**Published:** 2019-02-13

**Authors:** Jesús Pestana, Michael Maurer, Daniel Muschick, Manuel Hofer, Friedrich Fraundorfer

**Affiliations:** ^1^ Institute for Computer Graphics and Vision (ICG) Graz University of Technology (TU Graz) Graz Austria; ^2^ BIOENERGY2020+ GmbH Graz Austria

**Keywords:** aerial robotics, computer vision, mapping, planning

## Abstract

Achieving the autonomous deployment of aerial robots in unknown outdoor environments using only onboard computation is a challenging task. In this study, we have developed a solution to demonstrate the feasibility of autonomously deploying drones in unknown outdoor environments, with the main capability of providing an obstacle map of the area of interest in a short period of time. We focus on use cases where no obstacle maps are available beforehand, for instance, in search and rescue scenarios, and on increasing the autonomy of drones in such situations. Our vision‐based mapping approach consists of two separate steps. First, the drone performs an overview flight at a safe altitude acquiring overlapping nadir images, while creating a high‐quality sparse map of the environment by using a state‐of‐the‐art photogrammetry method. Second, this map is georeferenced, densified by fitting a mesh model and converted into an Octomap obstacle map, which can be continuously updated while performing a task of interest near the ground or in the vicinity of objects. The generation of the overview obstacle map is performed in almost real time on the onboard computer of the drone, a map of size 100m×75m is created in ≈2.75min, therefore, with enough time remaining for the drone to execute other tasks inside the area of interest during the same flight. We evaluate quantitatively the accuracy of the acquired map and the characteristics of the planned trajectories. We further demonstrate experimentally the safe navigation of the drone in an area mapped with our proposed approach.

## 1 INTRODUCTION

The utilization of drone technology in civilian applications is being limited by the requirement for drone operations to have a human pilot to ensure collision avoidance at all times. From a technical standpoint, first, most drones are not equipped with obstacle‐sensing technologies. And second, drone‐automated flight tends to make strong assumptions about the absence of obstacles along the flight route, for instance, during the takeoff and landing operations and more generally when flying close to the ground, buildings, and trees; hence, the requirement in practice for a pilot to ensure obstacle avoidance during flight. These are currently limiting factors for the automated operation of drones in promising high‐value operations, such as infrastructure inspection and package delivery. In addition, due to this current lack of automatic obstacle avoidance capabilities, setting up a fully automated flight in environments from which the operator has limited information, for instance, for search and rescue and disaster relief operations, is not feasible.

Early works on this topic motivated by the International Aerial Robotics Competition (IARC) Missions 3 and 4 (AUVSI Association, 2018) running, respectively, on years 1998–2000 and 2001–2008 showed promising results using unmanned helicopters and computer vision. In Mission 3, the aerial robot had to detect and avoid obstacles, identify survivors, and recognize drum containers. The winning team from the Technical University of Berlin (Kondak & Remuß, 2007; Musial, Brandenburg, & Hommel, 2000) was able to perform the target identification and localization tasks; however, their helicopter did not fly near the debris, but rather flew high over the area (Greer, McKerrow, & Abrantes, 2002). In Mission 4, the aerial robot had to identify a particular building and deploy a rover to accomplish a task inside it. A team of the Georgia Institute of Technology won this challenge (Johnson, Mooney, & Christophersen, 2013; Rooz et al., 2009) by completing the entire mission. Working in topics that relate to our presented work, the same team also developed a helicopter system able to fly over an area and acquire an accurate three‐dimensional (3D) reconstruction using a pan‐tilt‐mounted laser range finder (LADAR or LIDAR) and explored the 3D obstacle avoidance problem in simulation (Geyer & Johnson, 2006). A comparison of the 3D reconstructions obtained by performing an overview flight and acquiring and processing data from either a LIDAR or a camera is discussed on the work by Leberl et al. (2010). The IARC Mission 5 (2009) proposed the challenge of autonomously exploring an indoor area with tight spaces while searching for a target object on a wall. Mission 5 was fully accomplished using a quadrotor drone and a low‐weight LIDAR and a stereo camera system as main sensors by a team from the Massachusetts Institute of Technology (MIT; Bachrach, Prentice, He, & Roy, 2011). The same authors demonstrated similar capabilities using only LIDAR and a smaller drone platform in their work (Bachrach, He, & Roy, 2009).

**Figure 1:**
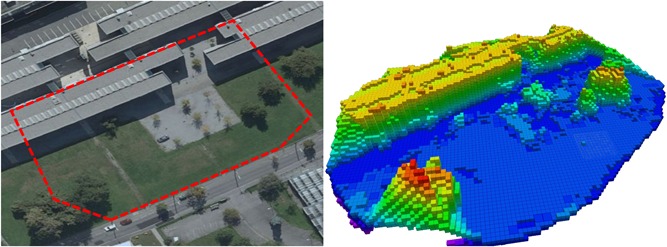
Obstacle map of an outdoor environment of size 105 m × 75 m generated from 56 images, with 12 m high buildings and up to 14 m high trees, generated using our method explained in Section 3.1. The obstacle map is displayed color‐coded according to the height and has a minimum voxel resolution of 1 m. On the left, “Google Earth ©2015,” an overview image of the area is shown, where the target 50 m × 50 m region of interest is located around the parking lot and a red contour denotes the effectively mapped area. The onboard processing time for the creation of this obstacle map was ≈2.75min. Man‐made obstacles, such as buildings and cars, and big trees are quite well reconstructed and included in the obstacle map. However, small trees are often not correctly mapped and need to be sensed later on during lower altitude flight. For this purpose, our drone, see Figure 2, is equipped with several stereo‐heads that can acquire point‐clouds of trees along with unmapped and dynamic obstacles during flight, which can be used to update the map [Color figure can be viewed at wileyonlinelibrary.com]

**Figure 2:**
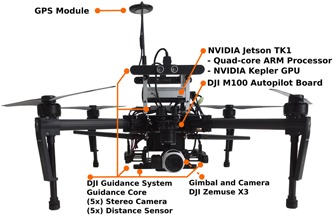
Hardware setup: DJI M100 drone, autopilot, GPS, gimbal camera, DJI Guidance system with five stereo camera heads, and a Nvidia Jetson TK1 onboard computer. GPS: global positioning system; GPU: graphics processing unit [Color figure can be viewed at wileyonlinelibrary.com]

As these early works show, the fast deployment of autonomous drones in unknown outdoor environments is since several years an ongoing research problem. In this study, some of the main challenges related to this topic are tackled, namely the acquisition of a good‐quality obstacle map and the calculation of trajectories that allow fast navigation in the area of interest. Our presented approach uses only onboard computation power, and as a result, the drone does not need to transfer data to a ground‐station via a wireless communication link. Our vision‐based mapping approach consists of two separate steps. First, the drone performs an overview flight at a safe altitude acquiring overlapping downward‐looking images, while creating a high‐quality map of the environment by using a state‐of‐the‐art photogrammetry method, the online Structure from Motion (SfM) pipeline (Hoppe et al., 2012; Rumpler et al., 2016). Second, this map is georeferenced and converted into an Octomap, see Figure 1, which is used as an initial overview obstacle map that can be updated during the rest of the flight while performing a task of interest near the ground. The generation of the overview obstacle map is performed in a few minutes on the drone onboard computer, and thus, with enough time remaining for the drone (Figure 2) to execute other tasks inside the area of interest during the same flight.

Our trajectory planning approach is designed to provide smooth trajectories away from obstacles. We have tested our navigation and trajectory planning algorithms, experimentally utilizing an obstacle map obtained using our mapping method. The aim of the experiment is to demonstrate the feasibility of autonomously deploying drones in unknown outdoor environments.

The concept of overview obstacle maps and the presented solution for drone deployment were inspired by the objectives of the *2016 DJI Developer Challenge*.^1^ This competition consisted of a search and rescue mission, in which the drone needed to explore a designated area searching for survivors. Our team, the *Graz Griffins*, took part in this challenge and was among the few participants that qualified to participate in the finals, where we demonstrated our solution at work.

The outline of this study is the following. The related work and our contributions are discussed in Section 2. The algorithms utilized for the realization of this study are described in Sections 3 and 4: the mapping approach in Sections 3.1 and 3.2, the navigation control in Section 4.1, and our trajectory planning solution in Section 4.2. The experiments are described and discussed in Section 5 with: the evaluation of the trajectory planner in Section 5.2, a qualitative and quantitative evaluation of the capabilities of our overview obstacle maps in Section 5.3, and an experimental flight showcasing performance of our system in a map acquired using our proposed approach in Section 5.4. Sections 6 and 7 contain the conclusions and a discussion about possibilities for future work.

## 2 STATE OF THE ART

Generating maps of areas of medium size in real‐time onboard a drone or leveraging offboard computing resources is a challenging task, and much research has been dedicated to it with varying degrees of success. The swarm of micro flying robots (SFLY) project (http://www.sfly.org/) (Scaramuzza et al., 2014) developed several novel algorithms for drones. A real‐time loosely coupled visual‐inertial odometry (VIO) framework, by Weiss, Achtelik, Lynen, Chli, and Siegwart (2012), was developed based on a modified version of parallel tracking and mapping (PTAM) (Klein & Murray, 2009) improved for onboard execution and on a computationally fast estimation algorithm used as a fall‐back method fusing the inertial measurement unit (IMU) readings with optical flow, thus only requiring a minimal amount of feature correspondences in consecutive frames. Using this, efficient version of PTAM (Weiss, Achtelik, Kneip, Scaramuzza, & Siegwart, 2011) showed an effective terrain exploration technique for micro‐aerial vehicles (MAVs) that generate, in real time in a ground‐station, a textured 3D mesh by means of a Delaunay triangulation (Labatut, Pons, & Keriven, 2007), which supports the drone operator in understanding the MAV’s environment.

Several research works have focused on the creation of maps that can be later reused by the drone to localize in real time during an autonomous flight. Surber, Teixeira, and Chli (2017) use the VIO algorithm open keyframe‐based visual‐inertial SLAM (OKVIS) (Leutenegger et al., 2013, 2015) to acquire a map of an area during a manual flight, and later reuse this map to reduce the UAV’s dependency on global positioning system (GPS) and evaluated their system against ground‐truth position data acquired with a Leica Total Station. Recently, researchers from the ETH Zürich have released a visual‐inertial mapping framework to process and produce multisession maps (T. Schneider et al., 2018), which uses robust visual inertial odometry (ROVIO) (Bloesch, Omari, Hutter, & Siegwart, 2015) as the VIO front‐end, and has been used to achieve autonomous drone flight (Burri, Oleynikova, Achtelik, & Siegwart, 2015). In Burri et al. (2015), the full bundle adjustment (BA) result and the obstacle map are generated after a manual flight and are later used in autonomous flights achieving precise indoor localization, navigation, and obstacle avoidance. The known state‐of‐the‐art visual SLAM frameworks ORB‐simultaneous localization and mapping (SLAM) (Mur‐Artal, Montiel, & Tardós, 2015) and ORB‐SLAM2 (Mur‐Artal & Tardós, 2017) also provide the capability of reusing a map acquired during a previous session or experiment. In Qiu, Liu, and Shen (2017), the authors propose the usage of mesh models obtained using well‐accepted off‐line SfM algorithms (Triggs, McLauchlan, Hartley, & Fitzgibbon, 1999) to substitute the usage of GPS. To achieve the vision‐based localization against the model, the authors propose an edge alignment scheme for the current image against a virtual image extracted from the model that is used as a reference or keyframe. A visual odometry framework (Schenk & Fraundorfer, 2017) using a similar algorithm for RGB‐D sensors provides a better evaluation on the approach and produces estimates with a drift accumulation on par with state‐of‐the‐art visual odometry (VO) methods.

Achieving dense mapping onboard a drone is very challenging due to the limited computational capabilities of their onboard computers. Several methods have been proposed that are too computation intensive and require powerful graphics processing units (GPUs), but the achieved levels of detail, and thus, the high quality of their dense maps would be extremely desirable for navigating drones. Examples of such dense‐reconstruction algorithms are the following: KinectFusion (Newcombe, Izadi, et al., 2011), ElasticFusion (Whelan, Leutenegger, Salas‐Moreno, Glocker, & Davison, 2015, 2016), MonoFusion (Pradeep et al., 2013), and dense tracking and mapping (DTAM; Newcombe, Lovegrove, & Davison, 2011), or the similar approach by Stühmer, Gumhold, and Cremers (2010). There is, thus, interest in the community in developing algorithms with better computational efficiency and accuracy trade‐off that could be executed onboard drones. Heng, Lee, Fraundorfer, and Pollefeys (2011) propose a method to generate a real‐time dense‐reconstruction offboard while guiding the drone by means of onboard VO. A framework to assist a surveyor while acquiring an SfM data set was proposed by Hoppe et al. (2012), an offboard calculated color‐coded mesh model is displayed in real time for the purpose of providing the surveyor with feedback about the local quality of the reconstruction. Wendel, Maurer, Graber, Pock, and Bischof (2012) utilize the drone onboard PTAM‐based calculated poses in an offboard server to produce a life dense 3D reconstruction that is displayed in real time on a tablet. The dense monocular 3D reconstruction algorithm regularized monocular depth estimation (REMODE) (Pizzoli, Forster, & Scaramuzza, 2014) measures depth against a reference view and performs uncertainty‐dependent point‐cloud smoothing achieving real‐time execution using CUDA (https://en.wikipedia.org/wiki/CUDA) by combining an algorithm to generate dense point‐clouds using patch‐level or per‐pixel Bayesian depth estimation using a parametric model (Vogiatzis & Hernández, 2011) and the fast state‐of‐the‐art visual odometry method semidirect visual odometry (SVO; Forster, Pizzoli, & Scaramuzza, 2014). REMODE has since been utilized on data acquired with drones to generate dense depth maps in real time for various research projects: creating medium‐sized maps in an offboard ground‐station by streaming the acquired data (Faessler et al., 2016), the creation of dense maps onboard (Forster, Faessler, Fontana, Werlberger, & Scaramuzza, 2015) by restricting their size to a relatively small 2.5D fixed‐size grid‐map around the robot (Fankhauser, Bloesch, Gehring, Hutter, & Siegwart, 2014), and the feasibility of sharing the 2.5D map acquired by the drone to guide a ground robot. Regarding the latter and still using REMODE, a mobile robot plans and executes trajectories in rough terrain in a small area mapped by a drone (Delmerico, Mueggler, Nitsch, & Scaramuzza, 2017), by training a terrain classifier on‐the‐fly (Delmerico, Giusti, Mueggler, Gambardella, & Scaramuzza, 2016), and a legged robot and the drone achieve localization on the same global coordinate frame in Käslin et al. (2016). In Lynen et al. (2015), an efficient indexed nearest‐neighbor search to achieve image‐based relocalization on a prebuilt map is proposed, where the map is obtained using standard SfM techniques with its scale recovered using IMU data and the recursive nonlinear filtering approach OKVIS (Agarwal et al., 2012; Leutenegger et al., 2013, 2015), and a VIO method for local pose tracking inspired on the multi‐state constraint kalman filter (MSCKF) (Mourikis et al., 2009) is used. Using OKVIS (Leutenegger et al., 2013, 2015) as VIO front‐end, the research work (Oleynikova, Burri, Lynen, & Siegwart, 2015) also proposes a method to localize a drone and a ground robot on the same map by means of a previously acquired reference map. Recently, a drone dense‐reconstruction (Karrer, Kamel, Siegwart, & Chli, 2016) data set has been released, which focuses on small working areas and producing precise 3D dense models for the purpose of object manipulation, in which ground‐truth position data acquired with a Leica Total Station are available.

The system developed by J. Schneider et al. (2016) creates a relatively dense georeferenced point‐cloud of very high accuracy while localizing the drone in real time at 100 Hz using only onboard computation on a 3.6 GHz Intel CPU (Santa Clara, CA) with 4 cores. Another possibility to create dense reconstructions is using VO semidense methods, which extract the depth of high‐gradient regions of the scene, such as large‐scale direct LSD‐SLAM (Engel, Schöps, & Cremers, 2014), Direct Sparse Odometry (DSO; Engel, Koltun, & Cremers, 2018), or semidense mapping (SDM; Mur‐Artal & Tardós, 2015). However, these methods are not well suited for this purpose because their depth estimates are not filtered or optimized for dense mapping. The direct tracking and mapping method dense piecewise‐planar tracking and mapping (DPPTAM; Concha & Civera, 2015) achieve good results by including piecewise‐planar surfaces in the model, but the computation requirement is too high for direct onboard execution. The method by Teixeira and Chli (2016) is extremely fast but the produced mesh results include strong interpolations causing error in sharp‐edges, such as corners. A later method from the same authors (Teixeira & Chli, 2017) uses large‐scale direct monocular SLAM (LSD‐SLAM), super‐pixels, and filtering that eliminates most depth outlier estimates, and it achieves very competitive runtimes on an Intel‐i7 4700MQ/Intel‐i7 5557U/Intel NUC processor (Intel Corporation (Intel), Santa Clara, CA) that can be mounted onboard drones.

The trajectory planner presented in this study was designed using methods from the state of the art to deliver long and smooth trajectories on our overview obstacle maps and it is presented as a component of the developed system. The reader is here directed to work in the field of fast trajectory replanning that would allow the drone to explore unknown cluttered environments while flying near the obstacles. These types of planners are able to regenerate an obstacle‐free smooth trajectory at a high rate, for instance, the following recent works make computation efficiency improvements by using operations in an OcTree data structure (Chen, Liu, & Shen, 2016), a local multiresolution discretization (Nieuwenhuisen & Behnke, 2016), and local replanners (Oleynikova et al., 2016; Usenko, vonStumberg, Pangercic, & Cremers, 2017).

In this study, we propose a method to create an overview obstacle map of a desired outdoor area onboard the drone. The success of our approach is a direct consequence of utilizing a survey flight trajectory that provides an image data set of a large area with high and approximately constant image overlap resulting in a very well constrained BA problem. This choice reduces the size of the optimization problem, for which the associated Hessian matrix has a known structure (Triggs et al., 1999), and keeps it at an onboard computationally manageable size. The resulting sparse 3D model is meshed to generate an obstacle map by using a Delaunay triangulation (Labatut et al., 2007). Although our approach comes at the cost of only mapping the obstacles that are well represented by the sparse SfM model, consisting of 3D points and lines, our densification operation is computationally very efficient. When compared with related work, our approach presents several novelties.

In comparison to previous work that re‐utilizes a map acquired on an earlier session for navigation (e.g., Burri et al., 2015; Qiu et al., 2017; T. Schneider et al., 2018), our solution allows the acquisition of a moderately sized obstacle map onboard the drone, which we demonstrate for maps of size 100 m × 75 m that are created in ≈2.75min, allowing the drone to perform a near‐ground navigation task on the same flight. Because the creation of our sparse 3D model is incremental, the mapping operation can be stopped at any time resulting in a smaller mapped area and a shorter map creation time. Similarly, in comparison to the discussed onboard dense‐reconstruction methods, our maps cover much bigger areas than the onboard solutions from the related work (e.g., Forster et al., 2015; Teixeira & Chli, 2017). Accuracy evaluation of our obstacle map is performed to provide a basis for the comparison of our obstacle map to that of other methods. Here, it is noted that in some works (e.g., J. Schneider et al., 2016), the accuracy is evaluated based on the distance of the mapped points to the ground‐truth point‐cloud rather than the other way around, which is not as informative for the purpose of using the 3D reconstruction as an obstacle map.

Similarly to Weiss et al. (2011), we densify the sparse model representation into an obstacle map by fitting a mesh model by using a Delaunay triangulation (Labatut et al., 2007), which is computationally very efficient. In comparison to the work by Weiss et al. (2011), we use (a) a conventional photogrammetry pipeline that reconstructs points and lines rather than an efficient version of PTAM and (b) a survey flight trajectory for the image acquisition, which together should result in a more accurate mesh model. In addition, our experiments provide new insights on the usage of the Delaunay triangulation from a sparse SfM model to create an obstacle map by (a) providing an accuracy evaluation of the generated mesh models using as ground‐truth dense point‐clouds obtained using a photogrammetry method and (b) demonstrating the usage of the calculated overview obstacle map for autonomous navigation.

In a nutshell, the main contributions of this study are as follows:

- We propose the concept of overview obstacle map generation for the fast deployment of drones in unknown outdoor environments. A short survey flight provides the data for the vision‐based incremental generation of the obstacle map onboard and leaves enough time to directly exploit the created map for near‐ground navigation.
- An accurate** evaluation of the generated obstacle map is presented in Section 5.3.
- We demonstrate the potential of our solution by performing autonomous obstacle‐free navigation on a map acquired using our proposed method.

## 3 OVERVIEW OBSTACLE MAP GENERATION

The calculation of the overview map is performed using an SfM algorithm. This choice allows us to use a minimal set of sensors commonly available on drones: a GPS sensor, used for scaling and georeferenciation, and a standard camera. In comparison to visual‐inertial approaches, we forgo the intersensor calibration of the camera with respect to the IMU sensor and the time synchronization of the data from both sensors, which could be achieved, for instance, using the *Kalibr* open‐source library from Furgale, Rehder, and Siegwart (2013). Additionally, SfM algorithms do not require a high frame rate and work well with still images, so that they can be used without a navigation computer vision camera. These characteristics allow the use of our solution in a broad range of commercially available drones.

### 3.1 Online SfM

The multirotor autonomously performs an overview flight at a safe high altitude over the region of interest. This region is defined by its GPS corner points. The drone takes off and ascends to a safe height, approaches the region of interest, and plans and executes a regular survey flight trajectory according to a desired image overlap setting. The set of acquired overlapping nadir images is used to generate a sparse 3D map enhanced with line features on‐the‐fly. Simultaneously, a 3D mesh representing the surface model is fitted to the sparse model and at the end of the survey flight, it is rasterized to obtain the overview obstacle map.

The utilized online SfM pipeline, developed at our institute,^2^ was first proposed by Hoppe et al. (2012), and it is very runtime efficient. The task of the online SfM is to reconstruct the scene 3D points and simultaneously calculate the camera poses against the calculated sparse point‐cloud. The pipeline is based on a precalibrated camera model that we obtained with the method of Daftry, Maurer, Wendel, and Bischof (2013) and utilizes scale invariant feature transform (SIFT) features (Lowe, 2004; Wu, 2007) to be able to handle imagery with large baselines. The sparse model is initialized from the first two images, for which a valid relative pose estimate can be computed by using the robust version of the five‐point pose estimation algorithm (Nistér, 2004). Afterward, the absolute pose estimation method by Kneip, Scaramuzza, and Siegwart (2011) is used to align the incoming images to the current sparse 3D reconstruction in real time. Meanwhile, iterative bundle adjustment (Triggs et al., 1999) is performed in a parallel thread to prevent the scene drift likely to be caused by the incremental map building procedure. Using an incremental and real‐time version of Line3D + + (Hofer, Maurer, & Bischof, 2017), a set of 3D lines is calculated from the aligned images.

The sparse 3D model, thus, consists of a point‐cloud, a set of line segments and the camera pose. The calculated set of 3D lines is sampled so as to add points and information for the calculation of the surface model. In addition, the lines enhance the interpretability of the visualization of the sparse 3D map, especially for man‐made structures and line‐rich regions.

Following the method (Rumpler et al., 2014, 2016), the GPS measurements and the camera poses of the sparse model are aligned by means of a robust random sample consensus (RANSAC)‐based least‐squares minimization of the distance between both sets of locations. This effectively provides the correct scale, rotation, and relative translation for the sparse 3D map. A better georeferenciation could be obtained by placing georeferenced fiducial markers on site; however, in this study, we forgo the use of markers so as to maintain our approach suitable for search and rescue scenarios.

In addition to the sparse 3D map, the pipeline also creates a surface model on‐the‐fly (Hoppe, Klopschitz, Donoser, & Bischof, 2013). The current implementation uses the approach by Labatut et al. (2007), rather than the approach outlined in Hoppe et al.’s (2013) work. This model is directly converted into an Octomap (Hornung, Wurm, Bennewitz, Stachniss, & Burgard, 2013) obstacle map representation by direct point‐sampling of the triangle mesh. The obtained Octomap can be updated in real time and is utilized by our trajectory planner during flight.

Because all these map representations of the environment are georeferenced, the drone can be localized in the map, with up to GPS precision, by simply using its internal GPS + IMU fusion provided by the autopilot board. Further, georeferenciation enables us to, first, add GPS‐defined no‐fly zones. And, second, it allows one to show the geolocation of objects of interest detected during flight.

**Figure 3:**
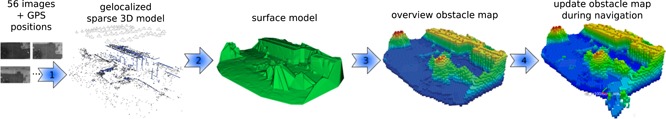
Successive steps undertaken to generate an obstacle map of size 105m×75m, see explanation in Section 3.1: autonomous overview flight at a safe altitude over the 50m×50m area of interest, generation of the sparse model using our incremental SfM pipeline, generating a dense surface model from the sparse one, conversion to Octomap by direct sampling of the surface model and successive updates during flight at low altitude by processing the stereo‐head data streams. The Octomaps are displayed color‐coded according to the height and have a minimum voxel resolution of 1 m [Color figure can be viewed at wileyonlinelibrary.com]

The experimental setup for the onboard real‐time execution of our mapping software is the following. The image stream from the Zenmuse X3 Gimbal (SZ DJI Technology Co., Ltd. (DJI), Shenzhen, Guangdong, China) (resolution of 1,280 × 720 pixels) is fully processed onboard our drone. This is made possible by leveraging the GPU of the onboard computer, an Nvidia Jetson TK1 development board, to extract image features using SiftGPU (Wu, 2007). The Online SfM pipeline is able to process one image every 3.0 s during flight experiments, or one image every 1.5 s when only processing a data set. A region of interest of size 50 m × 50 m with a final map size of up to around 105 m × 75 m (at ground‐level) is mapped onboard in ≈2.75min (Figure 1). This processing time includes: the overview flight, the acquisition of images, the generation of the sparse 3D model, the 3D mesh generation, the georeferenciation, and the conversion to the obstacle map representation (Figure 3).

### 3.2 Obstacle map representation

We use the Octomap (Hornung et al., 2013) obstacle map representation for this purpose, which is an OcTree‐based volumetric map representation (Figure 1, right). Its implementation is open‐source^3^ and it is integrated to be easily used with the Robot Operating System (ROS)^4^. We selected it for various reasons: It is memory and runtime efficient and achieves real‐time execution onboard and it can represent general‐shaped obstacles. In our approach, the obstacle map is obtained from a single mesh model, for which Octomap is a good fit. In contrast, methods based on creating the surface model using Truncated Signed Distance Fields (TSDFs), such as Voxblox (Oleynikova, Taylor, Fehr, Siegwart, & Nieto, 2017), are better suited to be used with depth sensors, such as RGB‐D and stereo cameras.

To accelerate the calculation of obstacle‐free trajectories, we use a precalculated distance map that provides the clearance of any point in free space to its closest obstacle. An efficient implementation of such an algorithm for Octomap was developed by Lau, Sprunk, and Burgard (2013) and released open‐source as a library named “*DynamicEDT3D*: A library for Incrementally Updatable Euclidean distance transforms in 3D’^5^. Its main advantages are featuring constant access‐time, because its internal data structure storing the distance map is an array, and being capable of time‐efficient incremental updates.

For the specific case of our drone, see Figure 2, the point‐clouds provided by our stereo‐heads are of low resolution (320 × 240 pixels). For this reason, during flight, we are able to apply fast incremental updates to both, the obstacle map and the distance map, by using their native Application Programming Interfaces (API). We have tested the runtime of this operation on data sets and for an Octomap with a minimum voxel resolution of 1 m, the updates can be applied onboard in real time, at a frequency higher than 1 Hz.

## 4 NAVIGATION USING THE OVERVIEW OBSTACLE MAP

The navigation controller design and tuning is explained in Section 4.1. To achieve safe near‐ground navigation in cluttered environments, we implemented a trajectory planner (see Section 4.2) that generates trajectories at a configurable clearance distance from obstacles.

### 4.1 Navigation control

#### 4.1.1 System identification

The flight behavior of our drone was characterized by performing speed command step response identification tests. Based on our experimental data and understanding of the system, the dynamical behavior from velocity command to the velocity output is assumed to be described by a transfer function

(1) $$P\left(s\right)≔\frac{v\left(s\right)}{v_{c}\left(s\right)}=e^{-sT_{d}}\;\frac{V}{Ts+1},$$

where s is the Laplace variable and P(s) maps the Laplace transform v(s) of v(t), the actual speed of the drone, to the Laplace transform $v_{c}\left(s\right)$ of $v_{c}\left(t\right)$, the speed command. The parameters of our model are: V, the static gain; $T_{d}$, a pure delay; and T, the time constant.

A rough controller parameter tuning was calculated based on the resulting model and later experimentally improved.

#### 4.1.2 Feedforward control

The mathematical model for the dynamics from velocity command to real velocity can be used to determine a feedforward control action. This action takes knowledge about the future development of the desired velocity into account and would thus, in the absence of errors, lead to the drone following the desired trajectory exactly. Because all axes are considered separately, the following section restricts itself to the x‐axis; all other axes can be handled in the same way.

Assuming that the desired trajectory $x_{d}\left(t\right)$ is given by a smooth mathematical function. Then, the value of the desired position $x_{d}$ and all of its derivatives ${\dot{x}}_{d}=v_{x,d},{\ddot{x}}_{d}={\dot{v}}_{x,d}$, and so forth are known at each time instant t. For our dynamics model of the drone, the transfer function (1), the feedforward control command is

(2) $$v_{\text{c,ff}}\left(t\right)=\frac{T}{V}{\dot{v}}_{d}\left(t+T_{d}\right)+\frac{1}{V}v_{d}\left(t+T_{d}\right),$$

that is, we need to look “into the future” by $T_{d}$ seconds and have knowledge about the desired acceleration and velocity. In the absence of modeling errors and flight disturbances, this command would lead to the drone following the desired trajectory exactly, that is, $v\left(t\right)\equiv v_{d}\left(t\right)$.

#### 4.1.3 Feedback control

The feedforward control law alone does not guarantee that the drone will actually follow the trajectory in a real setting, even if the initial position matches the beginning of the trajectory exactly. We utilize a feedback loop controller, similar to the PID controller architecture, for the three linear coordinates and the yaw heading and utilizing both position and speed measurement feedback and references. The utilized measurement feedbacks are the position, x(t), and velocity, $\dot{x}\left(t\right)$, from the internal GPS + IMU fusion provided by the autopilot board. An example speed command, $v_{c}$, for the autopilot over one of the coordinate axes is

(3) $$v_{\text{c,fb}}\left(t\right)=K_{p}\left[x_{d}\left(t\right)-x\left(t\right)\right]+K_{d}\left[{\dot{x}}_{d}\left(t\right)-\dot{x}\left(t\right)\right],$$

where $K_{p}$ and $K_{d}$ are the controller tuning parameters and $\left\{x_{d}\left(t\right),{\dot{x}}_{d}\left(t\right),{\ddot{x}}_{d}\left(t\right)={\dot{v}}_{d}\left(t\right)\right\}$ is the reference trajectory.

#### 4.1.4 Overall control

Sections 4.1.2 and 4.1.3 are combined in a single control law, resulting in the following equation for each of our coordinate axes:

(4) $$\begin{array}{ccc} v_{c}\left(t\right) & = & v_{\text{c,ff}}\left(t\right)+v_{\text{c,fb}}\left(t\right)=\frac{T}{V}{\ddot{x}}_{d}\left(t+T_{d}\right)+\frac{1}{V}{\dot{x}}_{d}\left(t+T_{d}\right)\\ & & +K_{d}\left[{\dot{x}}_{d}\left(t\right)-\dot{x}\left(t\right)\right]+K_{p}[x_{d}\left(t\right)-x\left(t\right)]. \end{array}$$

In our experiments, the measurement feedback utilized by the controller are the position, x(t), and velocity, $\dot{x}\left(t\right)$, from the GPS + IMU fusion provided by the autopilot board.

The reference smooth trajectory is calculated in two steps. First, a trajectory specified through waypoints, and accompanying speed and acceleration plans are obtained using the speed planner explained in Section 4.2. And, second, a third‐order spline is fitted to the set of waypoints, times of passage, speeds, and accelerations. The resulting spline is the reference smooth trajectory for the controller, specified in Equation (4) as $\left\{x_{d}\left(t\right),{\dot{x}}_{d}\left(t\right),{\ddot{x}}_{d}\left(t\right)\right\}$.

### 4.2 Trajectory planning

The purpose of our trajectory planner is to allow fast and safe navigation along long trajectories in cluttered environments. The calculated path should, therefore, be smooth and keep clear of obstacles. Whenever a new goal position is received, a new path is delivered to the controller. Similar to the approach proposed by Richter, Bry, and Roy (2013), we use the differential flatness of the quadrotor dynamics (Mellinger & Kumar, 2011) to plan a smooth trajectory in 3D position coordinates without directly considering the system dynamics, and perform the following calculations separately: obstacle‐free path planning and subsequent generation of a smooth, continuous, and differentiable trajectory path.

**Figure 4:**
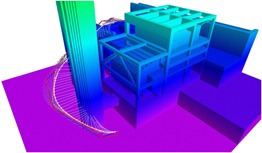
Synthetic outdoor Octomap environment of size 50 m × 50 m × 48 m. A 43.61‐m long planned trajectory obtained with our approach is shown. The path output of a state‐of‐the‐art planning algorithm (red) is consecutively shortened (green–blue–orange–white), resulting in the smoothed trajectory shown in white, which is further used to calculate a speed plan (semitransparent lines) and fit a spline that is used by the controller as trajectory reference. From the initial planning through all consecutive shortening operations, the trajectory optimizes an obstacle‐clearance metric, resulting in trajectories that provide a safe distance to nearby obstacles [Color figure can be viewed at wileyonlinelibrary.com]

Our method proceeds as follows (Figure 4):

1. Calculate a path using a state‐of‐the‐art trajectory planner that minimizes a cost function, which penalizes proximity to obstacles, unnecessary changes in altitude and length.
2. Limiting the increase on the path cost, the raw output path from the planning algorithm is consecutively shortened into a smooth trajectory.
3. Taking into account the path curvature and parameters that fix the maximum values for the velocity and acceleration, feasible time‐of‐passage over the waypoints, speed, and acceleration plans are calculated.
4. The resulting path, speed, acceleration, and timing information are used to fit a spline that is then used as trajectory reference and for the calculation of feedforward control commands by the navigation controller, see Section 4.1.

In the rest of this section, the intermediary steps of our trajectory planning approach are explained.

#### 4.2.1 Obstacle‐free path planning

The search for an obstacle‐free path is performed in 3D space without considering the attitude of the drone, which is set later by the acceleration plan based on the differential flatness property of the quadrotor dynamics (Mellinger & Kumar, 2011). The trajectory queries are from the current pose estimate, as a starting point, to the goal position.

A cost function is evaluated over candidate paths, see Equations (5)–(10), and is utilized to guide the search of the optimal path using a state‐of‐the‐art trajectory planning method. We have tested our approach using two different state‐of‐the‐art trajectory planning algorithms: the Probabilistic RoadMap (PRM; Kavraki, Svestka, Latombe, & Overmars, 1996) based algorithm ${\text{PRM}}^{*}$ (PRMStar; Karaman & Frazzoli, 2011) and the Rapidly exploring Random Tree (RRT; Lavalle, 1998) based algorithm ${\text{RRT}}^{*}$ (RRTStar; Karaman & Frazzoli, 2011) from the open motion planning library (OMPL) library (Şucan, Moll, & Kavraki, 2012).

Calculating the distance from multiple points of the trajectory to obstacles represents a computation bottleneck for any trajectory planning method. For this reason, we utilize the distance map library “DynamicEDT3D” proposed by Lau et al. (2013) in our implementation to accelerate the retrieval of the obstacle clearance, which is defined as the distance from a point to its closest obstacle, and for collision checking related calculations.

At this point, the trajectory is defined as a sequence of states, $s_{i}\in R^{3}$, joined by straight path segments, $l_{i}$. The path cost function, $c_{path}$, is the sum of its corresponding state, $c_{state}\left(s_{i}\right)$, and segment, $c_{segment}\left(l_{i}\right)$, costs. The segment cost is evaluated as the curvilinear integral of a cost per unit length function evaluated by sampling points over the segment, $x\in R^{3}$. Our cost penalizes length, proximity to obstacles and changes in the altitude of the trajectory. The path cost, $c_{path}$, is calculated as follows, see Equations (5)–(10):

(5) $$c_{path}=\sum_{s_{i}\in S}c_{state}\left(s_{i}\right)+\sum_{l_{i}\in L}c_{segment}\left(l_{i}\right),$$

(6) $$c_{state}\left(s_{i}\right)=c_{state}\left(x\right)=0,$$

(7) $$c_{segment}\left(l_{i}\right)=\int_{l_{i}}\left\{K_{c}\;c_{clearance}\left(x\right)+K_{a}\;c_{altitude}\left(x\right)+K_{l}\;c_{length}\left(x\right)\right\}\;|dl|,$$

(8) $$c_{clearance}\left(x\right)={\left\{1+K_{c2}\;\left(1-d_{clearance}\left(x\right)∕d_{max}\right)\right\}}^{4},$$

(9) $$c_{altitude}\left(x\right)=|u_{z}\left(x\right)|,$$

(10) $$c_{length}\left(x\right)=1,$$

where $K_{c},K_{c2},K_{a}$, and $K_{l}$ are tuning constants that set the relative strength of each cost contribution, $d_{max}$ is the maximum clearance distance at which the distance map is saturated, $d_{clearance}\left(x\right)$ is the value of the clearance provided by the distance map at $x,u_{z}\left(x\right)$ is the z component of the unitary vector along the path at x. In this study, the X and Y world axes are horizontal, and the Z‐axis is vertical and pointing upward. To prevent the planning algorithm from providing trajectories that traverse through obstacles, the clearance term, $c_{clearance}\left(x\right)$, introduces a cost of infinity when inside obstacles, that is, when $d_{clearance}\left(x\right)$ is 0.

The cost tuning constants are selected to achieve the following behavior. The trajectory is preferred to, in this order: not be unnecessarily near obstacles, not have unnecessary changes in altitude, and be as short as possible. The resulting trajectory is, rather than the global optimum, the feasible path of minimum cost, as defined by Equations (5)–(10), among those explored by the trajectory planning routine during a preset amount of time.

#### 4.2.2 Trajectory shortening and smoothing

The resulting raw path from the prior step usually presents sharp angles at many waypoints. Therefore, in this step, the path is modified by performing a sequence of obstacle‐aware shortening and smoothing operations. The blind application of these operations would result in a path, which would pass too near obstacles, corresponding to a numerically high cost for the smoothed path in comparison to the raw path.

To avoid this, some increase in the path cost is allowed, but it is constrained to a fraction of the raw path cost. In this manner, the above‐mentioned sought qualities of the raw path are kept in the smoothed path. The shortening and the smoothing are the result of applying subsequent suboperations iteratively, for which a cost increase can be calculated individually. Therefore, the performed path simplifications are all cost‐aware, that is, a shortening or smoothing suboperation is only accepted when its corresponding cost increase, measured by means of Equations (5)–(10), is below a threshold.

The performed suboperations are the following (Figure 4):

1. Reduce the number of vertices that are present in the current path: Interim waypoints are removed if the trajectory is still collision‐free and until the total cost does not increase more than 10%.
2. Collapse waypoints that are too near each other, an overall allowed cost increase of 10%.
3. Shortcut the path, an allowed cost increase of 10%: Not only waypoints are considered for the path length reduction, but also inner points of the path segments.
4. Smooth the path using the B‐spline algorithm with an allowed cost increase of 15%: New waypoints are sampled making the path rounder around sharp corners.
5. New waypoints are sampled in the current trajectory segments, and others are reduced to achieve segment lengths inside an acceptable predefined range, an allowed cost increase of 10%.

The resulting smooth path sets the final position coordinates for all the waypoints. In the next step, only the dynamic information of the trajectory, for example, speed and times of passage, is calculated.

#### 4.2.3 Speed, acceleration, and time‐of‐passage planning

We are interested in being able to explicitly set maximum speed and acceleration constraints. Our approach achieves this by taking inspiration in the work of Hoffmann, Waslander, and Tomlin (2008) and making improvements to it, so as to produce a smoother speed plan, for example, with more continuous acceleration derivatives.

The main configuration parameters of the algorithm consist of the maximum upward, downward, and horizontal velocities and accelerations, $\left\{v_{max,h},v_{max,z,up},v_{max,z,down},a_{max,h},a_{max,z,up},a_{max,z,down}\right\}$. These parameters define the velocity saturation constraints as the ellipsoids $((v_{x}^{2}+v_{y}^{2})/v_{max,h}^{2})+(v_{z}^{2}/v_{max,z,up}^{2})=1$ and $((v_{x}^{2}+v_{y}^{2})/v_{max,h}^{2})+(v_{z}^{2}/v_{max,z,down}^{2})=1$. The acceleration constraints are similarly defined. In Algorithms 1 and 2, the maximum values for the speed and the acceleration provided by the four ellipsoids are retrieved by the functions Velocity_Max_Direction(**d**, config) and Acceleration_Max_Direction(**d**, config).

The desired speed for the trajectory is set at every point to the minimum of the following two values: (a) the maximum configured velocity, Velocity_Max_Direction(…), and (b) the maximum attainable velocity as limited by the radius of curvature and the maximum acceleration, $v_{max,i}=\sqrt{a_{max,n}\;\cdot \;r_{i}}$, where $a_{max,n}\;=$ Acceleration_Max_Direction $\left(n_{i}^{*},\;\text{config}\right)$, with $n_{i}^{*}$ being the estimated normal vector to the trajectory at waypoint[*i*]. Then, the desired initial and the final velocities are set, being usually both set to zero.

The generation of the speed plan entails the following steps:

1. The tangent, normal and binormal vectors are estimated at each waypoint. Afterward, with this information, the position of the center and the radius of curvature are calculated by estimating the circumference that approximates each waypoint[*i*] and its neighbors. To perform this calculation, each point[*i*] and a sampling of its immediate neighbors along the path are projected into the plane formed by the current estimate of the normal and tangent vectors; and a system of equations is solved to determine the parameters of the said circumference. The radius of curvature at each waypoint and new estimates for the tangent and normal vectors are retrieved based on the solution to the system of equations.
2. A first speed plan is calculated that complies with the acceleration constraints. For this purpose, the algorithm Velocity_Plan_Sweep_Double_Pass(…) that was originally proposed by Hoffmann et al. (2008) is used with only minor modifications, see Algorithms 1 and 2. In this algorithm, the equations relating, for every section of the trajectory, to path segment lengths, velocities, and accelerations correspond with those of linear uniformly accelerated motion. The modifications to this algorithm are: (a) see line 3 of Algorithm 2, saturating for both normal and tangential accelerations and (b) allowing the saturation of velocities and accelerations differently depending on the direction. A comparison between the smoothed and nonsmoothed speed plans is done in Section 5.2.1.

**Table:** 

**Algorithm 1** Velocity_Plan_Sweep_Double_Pass ($s,r,{\left\{t_{i}^{*}\right\}}_{i},v_{desired}$, config)
1: $v_{plan}=v_{desired}$
2: $v_{init}\leftarrow v_{desired,1}$
3: $v_{end}\leftarrow v_{desired,N}$
4: $\text{flip}\left\{s,r,{\left\{t_{i}^{*}\right\}}_{i},v_{desired},v_{plan}\right\}:{∕}^{*}$ the flip function flips, or interchanges, the elements of each vector, so that they are timewise reversed */
5: s=(−1)⋅s
6: $\left\{v_{plan},a_{at,plan},\Delta t_{plan}\right\}=$ Velocity_Plan_Sweep $\left(v_{end},s,v_{plan},r,{\left\{t_{i}^{*}\right\}}_{i},\text{config}\right);{∕}^{*}$ see Algorithm 2 */
7: flip$\left\{s,r,{\left\{t_{i}^{*}\right\}}_{i},v_{desired},v_{plan}\right\}$
8: s=(−1)⋅s
9: $\left\{v_{plan},a_{at,plan},\Delta t_{plan}\right\}=$ Velocity_Plan_Sweep $\left(v_{init},s,v_{plan},r,{\left\{t_{i}^{*}\right\}}_{i},\text{config}\right)$
10: **return** $\left\{v_{plan},a_{at,plan},\Delta t_{plan}\right\};{∕}^{*}$ note that the cross‐track acceleration is $a_{ct,plan}={\left[v_{plan,i}^{2}∕r_{i}\right]}^{*}∕$

**Table:** 

**Algorithm 2** Velocity_Plan_Sweep($v_{init},s,v,r,{\left\{t_{i}^{*}\right\}}_{i},\text{config}$)
1: $v_{1}\leftarrow v_{init}$
2: **for** i=1 to N **do**
3: $a_{max}=Acceleration\text{\_}Max\text{\_}Direction\left(t_{i}^{*},\text{config}\right)-\frac{v_{i}^{2}}{r_{i}}$;
4: $a_{at,i}\leftarrow \text{min}\left(a_{max},\frac{v_{i}^{2}-{\left({\bar{v}}_{i+1}\right)}^{2}}{2\left(s_{i}-s_{i+1}\right)}\right)$
5: **if** $a_{at,i}>0$ **then**
6: $v_{i+1}\leftarrow \sqrt{v_{i}^{2}-2a_{at,i}\left(s_{i}-s_{i+1}\right)}$
7: $\Delta t_{i}\leftarrow \frac{-v_{i}+\sqrt{v_{i}^{2}-2a_{at,i}\left(s_{i}-s_{i+1}\right)}}{a_{at,i}}$
8: **else**
9: **if** $v_{i}>{\bar{v}}_{i+1}$ **then**
10: $v_{i+1}\leftarrow {\bar{v}}_{i+1}$
11: $a_{at,i}\leftarrow \frac{v_{i}^{2}-v_{i+1}^{2}}{2\left(s_{i}-s_{i+1}\right)}$
12: $\Delta t_{i}\leftarrow \frac{-v_{i}+\sqrt{v_{i}^{2}-2a_{at,i}\left(s_{i}-s_{i+1}\right)}}{a_{at,i}}$
13: **else**
14: $v_{i+1}\leftarrow v_{i}$
15: $a_{at,i}\leftarrow 0$
16: $\Delta t_{i}\leftarrow \frac{\left(s_{i+1}-s_{i}\right)}{v_{i}}$
17: **end if**
18: **end if**
19: **end for**
20: **return** $\left\{v_{plan}=v,a_{at,plan},\Delta t_{plan}\right\}$; // note that the cross‐track acceleration is $a_{ct,plan}=\left[v_{plan,i}^{2}∕r_{i}\right]$

3. The output speed plan, $v_{plan}$, of the Velocity_Plan_Sweep_Double_Pass(…) function, Algorithm 1, complies with the configured velocity and acceleration constraints, considering both normal and along‐track accelerations. However, it often provides a bang–bang solution that proposes maximum acceleration values with opposite signs at certain consecutive waypoints of the path. For this reason, in the next step and in contrast to Hoffmann et al. (2008), we propose an iterative speed plan smoothing algorithm that results in a continuous acceleration plan with a feasible derivative. The velocity smoothing operation is summarized in Algorithm 3 and it consists of the following steps:
  (a) Using the current velocity plan as a data term, a new velocity plan is calculated applying a smoothing spline‐type optimization, see a related chapter of the book (James, Witten, Hastie, & Tibshirani, 2013). This corresponds to minimizing the following cost function, see Equations (11) and (12), by using the Gauss–Newton least‐squares minimization (Triggs et al., 1999), note that 1 and N are the indexes of the initial and end velocities:
    (11) $$\begin{array}{ccc} f\left(v,v_{old},\Delta t=\Delta t_{plan}\right) & = & \lambda_{1}\left[\overset{i=N-1}{\underset{i=2}{\sum }}\;{\left(v_{i}-v_{i,old}\right)}^{2}\right]+\lambda_{2}\left[\int_{t_{1}}^{t_{N}}{\left(\frac{d^{2}v}{dt^{2}}\right)}^{2}dt\right]\\ & & \;+\lambda_{3}[\int_{t_{1}}^{t_{N}}{\left(\frac{d^{3}v}{dt^{3}}\right)}^{2}dt], \end{array}$$
    (12) $$\begin{array}{ccc} & = & \lambda_{1}\left[\overset{i=N-1}{\underset{i=2}{\sum }}\;{\left(v_{i}-v_{i,old}\right)}^{2}\right]+\lambda_{2}\left[\overset{i=N-2}{\underset{i=1}{\sum }}\;\left\{{\left(\frac{a_{at,i+1}-a_{at,i}}{\Delta t_{T_{at,i}}}\right)}^{2}\Delta t_{T_{at,i}}\right\}\right]\\ & & \;+\lambda_{3}\left[\overset{i=N-3}{\underset{i=1}{\sum }}\;\left\{{\left(\frac{T_{at,i+1}-T_{at,i}}{{\Delta t}_{\Delta T,i}}\right)}^{2}{\Delta t}_{\Delta T,i}\right\}\right],\;\text{where}\;{\Delta t}_{T_{at,i}}\\ & = & \frac{\Delta t_{i+1}+\Delta t_{i}}{2}\;\text{and}\;{\Delta t}_{\Delta T,i}=\frac{\Delta t_{i+2}+2\Delta t_{i+1}+\Delta t_{i}}{4}. \end{array}$$
    In these equations,
    i. $a_{at,i}$, along‐track acceleration at waypoint i,
      (13) $$a_{at,i}=\frac{v_{i+1}-v_{i}}{\Delta t_{i}},$$
    ii. $T_{at,i}$, derivative of the along‐track acceleration at waypoint i,
      (14) $$T_{at,i}=\frac{\left(a_{at,i+1}-a_{at,i}\right)}{{\Delta t}_{T_{at,i}}}=\frac{\left(\frac{v_{i+2}-v_{i+1}}{\Delta t_{i+1}}-\frac{v_{i+1}-v_{i}}{\Delta t_{i}}\right)}{{\Delta t}_{T_{at,i}}}.$$
    The similarity of the smoothing cost function to the one minimized by a smoothing spline is shown in Equation (11), see related chapter of the book (James et al., 2013). The expression is first developed using the intermediary variables $a_{at,i}$ and $T_{at,i}$ resulting in Equation (12), and for the optimization, it is further developed to depend only on the speed plan using Equations (13) and (14). The data term, the old velocity plan $v_{old}$, and the time intervals between waypoints, $\Delta t=\Delta t_{plan}$, are held constant during each smoothing spline optimization iteration. The optimization is performed using the Gauss–Newton least‐squares, where only the current velocity plan is considered as an optimization variable v. The initial, $v_{1}$, and end, $v_{N}$, velocities are kept constant, and therefore, not optimized. The parameters $\left\{\lambda_{1},\lambda_{2},\lambda_{3}\right\}$ are weights adjusting the strength of each type of optimization residual: $\lambda_{1}$ regulates the strength of the data term, $\lambda_{2}$ regulates the strength of the acceleration smoothing terms, and $\lambda_{3}$ regulates the strength of the acceleration derivative smoothing terms. A set of parameter weights that have provided good results and that were used to obtain the values shown in the simulation and experimental tests are the following: $\lambda_{1}=300$, $\lambda_{2}=1.12$, and $\lambda_{3}=0.08$. Considering the notation from the SfM review paper (Triggs et al., 1999), the previously defined cost function, Equation (12), can be rewritten using residuals as follows, see Equations (15)–(20). In these equations, W is the weight matrix and Δz the residual vector. The residual vector is subdivided in the three types of cost, $\Delta z_{v_{0}}\Delta z_{a}\Delta z_{T}$, from the smoothing cost function, see Equations (11) and (12). $\Delta z_{v_{0},i}$ is defined for i=2,…,N−1. $\Delta z_{a,i}$ is defined for i=1,…,N−2. $\Delta z_{T,i}$ is defined for i=1,…,N−3.
    (15) $$f\left(v,v_{old},\Delta t=\Delta t_{plan}\right)=\frac{1}{2}\Delta z^{⊤}W\Delta z,$$
    (16) $$\Delta z=\left[\Delta z_{v_{0}},\;\Delta z_{a},\;\Delta z_{T}\right],$$
    (17) $$W=diag\left(\lambda_{1}I_{N-2},\lambda_{2}I_{N-2},\lambda_{3}I_{N-3}\right),$$
    (18) $$\Delta z_{v_{0},i}=(v_{i}-v_{i,old}),$$
    (19) $$\Delta z_{a,i}=T_{at,i}\sqrt{\Delta t_{T_{at,i}}}=\frac{\left(a_{at,i+1}-a_{at,i}\right)}{\Delta t_{T_{at,i}}}\sqrt{\Delta t_{T_{at,i}}},$$
    (20) $$\Delta z_{T,i}=\left(\frac{T_{at,i+1}-T_{at,i}}{{\Delta t}_{\Delta T,i}}\right)\sqrt{{\Delta t}_{\Delta T,i}}.$$
    Using this notation, the increment to the speed plan, $\Delta v_{c}$, at each iteration is calculated from the following set of equations $\left(J^{⊺}WJ\right)\Delta v_{c}=-J^{⊺}W\Delta z$, where *J* is the Jacobian matrix of Δz.
  (b) After every iteration of the smoothing spline optimization, the resulting speed plan is not self‐consistent. This means that the values $\left\{v_{plan},a_{at,plan},\Delta t_{plan}\right\}$ do not verify the equations of a uniformly accelerated motion for all segments of the trajectory. In fact, the smoothing spline optimization does not enforce these constraints. For this reason, after every iteration of the smoothing spline optimization, a new pass of Velocity_Plan_Sweep_Double_Pass(…) is performed, so that a new set of self‐consistent values for $\left\{v_{plan},a_{at,plan},\Delta t_{plan}\right\}$ is obtained. This amounts to the following function call $\left[v_{plan},a_{at,plan},\Delta t_{plan}\right]=\text{Velocity\_Plan\_Sweep\_}\text{Double\_Pass}\left(s,r,{\left\{t_{i}^{*}\right\}}_{i},v_{new\;max},\;\text{config}\right)$, where $v_{new\;max}$ is defined for every component as the minimum of $v_{plan}+\Delta v_{c}$ and $v_{desired}$, that is, $v_{new\;max}=\left\{v_{new\;max,i}=min\left(v_{plan,i}+\Delta v_{c,i},v_{desired,i}\right)\right\}$ (see Algorithm 3). The iterative Gauss–Newton least‐squares optimization that encodes the velocity smoothing algorithm, Algorithm 3 lines 4–11, is stopped when: (a) the norm of the Jacobian of the cost function, Equations (11) and (12), increased in the last iteration or (b) a preset number of iterations are reached.
  (c) Rerunning the smoothing spline optimization exchanging the data term by the last smoothed velocity plan, Algorithm 3 lines 1–12, allows the algorithm to gradually forget the strong initial bang–bang velocity plan provided by Algorithm 1. For a given set of the smoothing strength parameters $\left\{\lambda_{1},\lambda_{2},\lambda_{3}\right\}$, increasing the number of reruns of the smoothing spline optimization results in smoother speed plans, with lower maximum values for the acceleration derivative. This effect is demonstrated in Section 5. This fact allows the calculation of a speed plan with an acceleration derivative bounded by a specific value. In practice, to obtain efficient computation times, the smoothing spline optimization is repeated a fixed number of times.

**Table:** 

**Algorithm 3** Velocity_Smoothing($s,r,{\left\{t_{i}^{*}\right\}}_{i},v_{plan},\Delta t_{plan},r$)
1: **for** i=1 **to** num_passes **do**
2: $v_{old}=v_{plan}$
3: **for** j=1 **to** max_iterations_per_pass **do**
4: $\left\{\Delta v_{c},|\frac{df}{dv}{|}_{j}\right\}=$ velocity_smoothing_iteration$\left(v_{plan},v_{old},\Delta t_{plan},r\right)$
5: **if** $\left(\left(j==1\right)\mathrm{or}\left(|\frac{df}{dv}{|}_{j}<|\frac{df}{dv}{|}_{j-1}\right)\right)$
6: $v_{newmax}=min\left(v_{plan}+\Delta v_{c},v_{desired}\right)=\left\{v_{newmax,i}=min\left(v_{plan,i}+\Delta v_{c,i},v_{desired,i}\right)\right\}$
7: $\left[v_{plan},a_{at,plan},\Delta t_{plan}\right]=\text{Velocity\_Plan\_Sweep\_Double\_Pass}\left(s,r,{\left\{t_{i}^{*}\right\}}_{i},v_{newmax},\text{config}\right)$
8: **else**
9: **break**
10: **end if**
11: **end for**; ${∕}^{*}$ j */
12: **end for**; ${∕}^{*}$ i */
13: **return** $\left\{v_{plan},a_{at,plan},\Delta t_{plan}\right\};{∕}^{*}$ note that the cross‐track acceleration is $a_{ct,plan}={\left[v_{plan,i}^{2}∕r_{i}\right]}^{*}∕$
4. To calculate the trajectory reference for the navigation controller, a third‐order spline is fitted to the set of waypoints, times of passage, speeds, and accelerations, see Section 4.1. The spline representation allows the navigation controller to calculate the trajectory references at any instant in time.

### 4.3 Geometric speed planner

In Section 4.2, we have described a planning approach, which divides the trajectory generation in separate three subproblems: obstacle‐free path planning, trajectory shortening, and smoothing; and speed, acceleration, and time‐of‐passage planning. The presented speed planning approach can also be used when the drone flies in obstacle‐free areas over paths specified by manually defined waypoints. For this purpose, the “Geometric Speed Planner” module was developed, which implements only the following two subproblems: (a) trajectory smoothing that produces a path following the specified sequence of waypoints but that generates curves at the intermediary waypoints; and (b) the calculation of the speed, acceleration, and time‐of‐passage plans.

## 5 EXPERIMENTAL RESULTS

We have performed three types of experiments for the evaluation of our solution, the combination of the mapping procedure, see Sections 3.1 and 3.2, and our navigation approach, see Sections 4.1 and 4.2. In Section 5.2, the runtime and the characteristics of the trajectories generated by our planning approach have been benchmarked in a synthetic industrial environment. In Section 5.3, we showcase the quality of the generated overview maps in three different scenes and analyze quantitatively the accuracy of the onboard generated overview obstacle maps against an offboard state‐of‐the‐art dense‐reconstruction photogrammetry method. And in Section 5.4, the usage of overview obstacle maps for navigation is demonstrated on an autonomous flight experiment.

### 5.1 Experimental platform

The drone shown in Figure 2 was used in our experiments. It is equipped with a powerful onboard computer and several sensors. Its main equipment is:

- DJI M100 drone (SZ DJI Technology Co., Ltd. (DJI), Shenzhen, Guangdong, China), which, with a TB47D battery and equipped as described, features a takeoff weight of 3.5 kg and achieves a flight time of approximately 12 min.
- Nvidia Jetson TK1 (DJI Manifold, Nvidia Corporation (Nvidia), Santa Clara, CA) onboard computer that features a quad‐core processor, 2 GB of RAM and a CUDA‐enabled Tegra chip.
- Zenmuse X3 Gimbal camera: 1,280 × 720 pixels at 30 Hz.
- DJI Guidance visual‐sensing system with five stereo‐heads simultaneously provides images from all five directions and point‐clouds from two directions at 320 × 240 pixels at 10 Hz.

### 5.2 Evaluation of the trajectory planning approach

**Figure 5:**
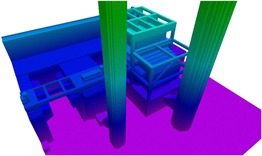
Synthetic obstacle map of size 50×50×48m used to benchmark the capabilities of our trajectory planner. The map features a fair amount of clutter and small passages with a width of 5.25 m [Color figure can be viewed at wileyonlinelibrary.com]

The evaluation of the trajectory planner is performed in a synthetic obstacle map (Figure 5) that represents an industrial environment of size 50×50×48m, which is distributed as part of the GitHub repository of the RotorS simulation framework (Burri, 2015; Furrer, Burri, Achtelik, & Siegwart, 2016). Using this environment, we showcase the capabilities of our trajectory planner to generate smooth trajectories around and away from obstacles.

The configuration of the trajectory planner in this section is as follows. The PRM* and the RRT* planners are configured with the default parameters from the OMPL library, except for the maximum distance limit of a new vertex to the current tree in the RRT* algorithm, which is deactivated. The obstacle‐free trajectory calculation was configured with a maximum distance $d_{max}$ of 6 m for the distance map and a maximum planning time of 1 s. This particular choice for the maximum planning time is based on the fact that the overview obstacle map contains abundant free space above ground. In most cases, the target point is reachable and the trajectory planning algorithm is able find at least one valid solution on the allotted time. Therefore, the resulting trajectory is the best solution that was found in 1 s. The utilized Octomap resolution is 0.25 m. The trajectory planner was set up with a maximum speed of 20.0 m/s, and maximum horizontal, upward, and downward accelerations of $0.5g=4.91{\text{m∕s}}^{2},0.45g=4.41{\text{m∕s}}^{2}$ and $0.4g=3.92{\text{m∕s}}^{2}$, respectively.

#### 5.2.1 Comparison of speed and acceleration plans with and without velocity smoothing

**Figure 6:**
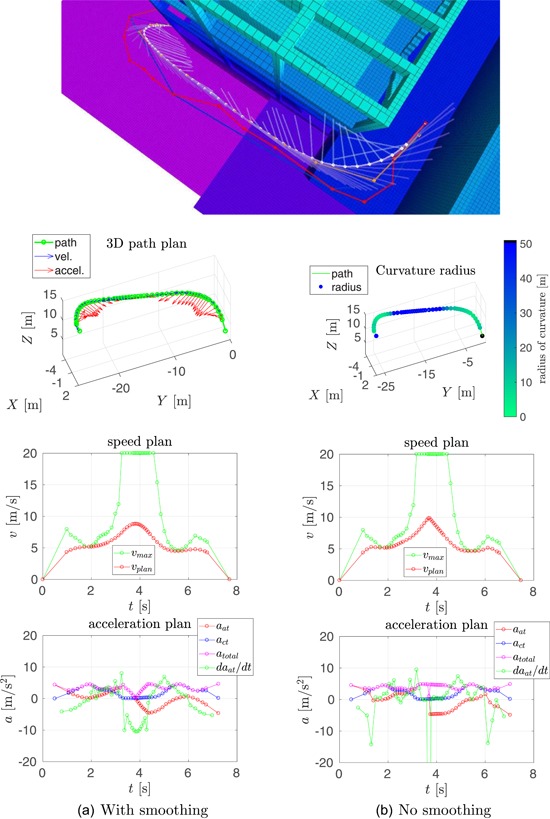
Example trajectory 1—Comparison of speed and acceleration plans with and without velocity smoothing. (top) Visualization of the planned trajectory. Regarding the rest of the plots: (top‐left) trajectory path in 3D, (top‐right) path color‐coded with the radius of curvature, (middle, bottom‐left) smoothed and (middle, bottom‐right) nonsmoothed speed, and acceleration plans (a) with smoothing and (b) no smoothing [Color figure can be viewed at wileyonlinelibrary.com]

**Figure 7:**
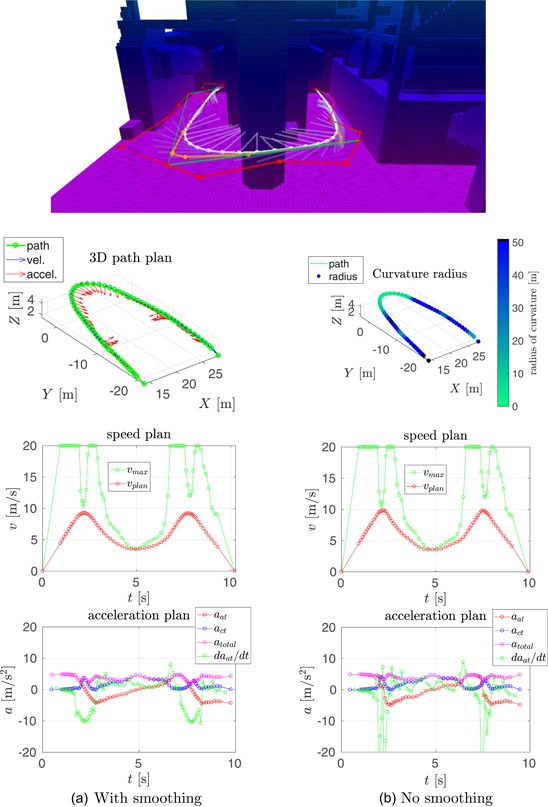
Example trajectory 2—Comparison of speed and acceleration plans with and without velocity smoothing. (top) Visualization of the planned trajectory. Regarding the rest of the plots: (top‐left) trajectory path in 3D, (top‐right) path color‐coded with the radius of curvature, (middle, bottom‐left) smoothed and (middle, bottom‐right) nonsmoothed speed, and acceleration plans (a) with smoothing and (b) no smoothing [Color figure can be viewed at wileyonlinelibrary.com]

In this section, we compare our speed planning method, see Section 4.2.3, to the one proposed by Hoffmann et al. (2008), from which our method was inspired. The result of running both methods in two trajectories is shown in Figures 6 and 7.

The following information is shown in these figures: (top) visualization of the planned trajectory with the Octomap displayed color‐coded for altitude from low (pink) to high (blue, green, and red). The (red) raw path planned by the state‐of‐the‐art trajectory planner is shown along with the consecutive path shortening and smoothing steps resulting in the (white) smoothed path. The semitransparent lines show the velocity and acceleration plans. The next two plots, inspecting the figure from top to bottom, show the trajectory path in 3D: at the (left), the waypoints are shown in green, along with the speed and the acceleration plans in blue and red, respectively; at the (right), the trajectory path is displayed color‐coded for the radius of curvature, where black color denotes parts of the trajectory estimated to be straight. The remaining four plots are grouped in vertical pairs, where the (top) plot shows the final along‐track speed plan in red, with the maximum desired velocity in green; and the (bottom) plot shows the along‐track, the cross‐track and total accelerations in red, blue, and magenta, respectively, along with the derivative of the acceleration in green. The maximum velocity constraint is a combination of the maximum velocity and the maximum acceleration constraints through the calculated radius of curvature and the required centripetal acceleration.

In the context of the speed and acceleration plans, on the (left), the result of applying the velocity smoothing optimization, and on the (right), the result of applying only the velocity_plan_sweep_double_pass, our implementation of the algorithm by Hoffmann et al. (2008), are presented. When compared with the method proposed by Hoffmann et al. (2008), our velocity smoothing approach results in a slightly higher trajectory traversal time with similar values for the maximum velocity but with much more feasible velocity and acceleration plans and with bounded and more continuous values for the acceleration derivative.

**Figure 8:**
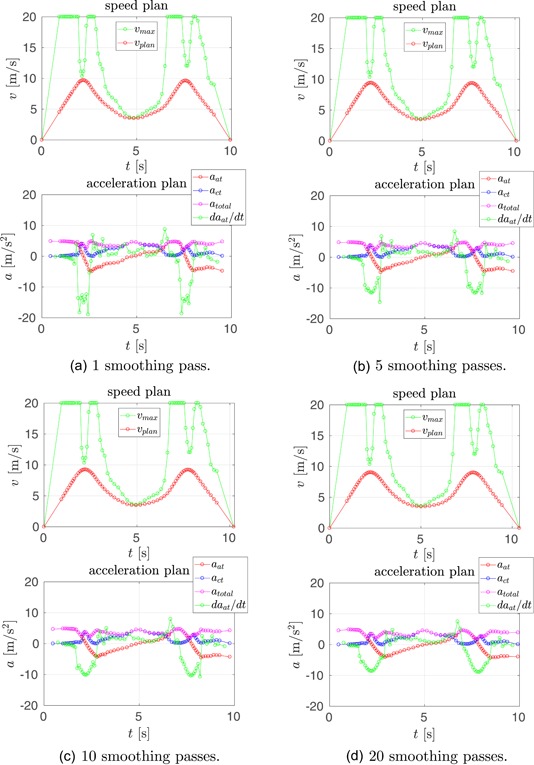
Example trajectory 2, see Figure 7—Comparison of speed and acceleration plans with a varying number of velocity smoothing passes. Each pair of plots shows the corresponding plans for a different number of passes, resulting in the following [number of passes, maximum acceleration derivative]: (upper left) $\left[1;\;19m/s^{3}\right]$, (upper right) $\left[5;\;15m/s^{3}\right]$, (down left) $\left[10;\;11m/s^{3}\right]$, and (down right) $\left[20;\;9m/s^{3}\right]$. The result of not applying any smoothing passes is shown in Figure 7 (middle, bottom‐right). (a) 1 smoothing pass, (b) 5 smoothing passes, (c) 10 smoothing passes, and (d) 20 smoothing passes [Color figure can be viewed at wileyonlinelibrary.com]

In Figure 8, a comparison of the resulting speed and acceleration plans when applying a varying number of velocity smoothing passes is shown. The plots have the same meaning as each pair of vertical plots in the lower part of Figures 6 and 7. As shown, for a given set of the smoothing strength parameters $\left\{\lambda_{1},\lambda_{2},\lambda_{3}\right\}$, increasing the number of reruns of the smoothing spline optimization results in smoother speed plans, with lower maximum values for the acceleration derivative.

The discussed results, see Figures 6–8, showcase the importance of utilizing our velocity smoothing approach and that by applying an increasing number of smoothing passes, the derivative of the acceleration becomes more continuous and achieves consecutively lower absolute values. Based on these results, in the rest of the evaluation and experiments, we use 10 passes for the speed plan smoothing.

#### 5.2.2 Performance benchmarking of the trajectory planner

**Figure 9:**
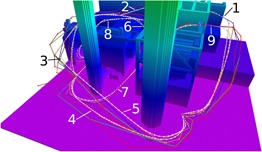
Example trajectories from the evaluation of the planner described in Section 5.2, color coding of the trajectories as explained in Figure 4 [Color figure can be viewed at wileyonlinelibrary.com]

The capabilities of our trajectory planning approach are evaluated by testing it on nine different queries repeatedly on the synthetic obstacle map, as shown in Figure 9. Each query corresponds to a given initial and target point. This evaluation was executed directly on the drone’s onboard computer, a Nvidia Jetson TK1 development board. In order for the repeated evaluation not to be dependent on the internal state of the planner, which is relevant when using the PRM* algorithm, the planner is reinitialized after querying the planner for the nine trajectories. This process was repeated 100 times using the PRM* and the RRT* algorithms, resulting on the performance statistics shown in Tables 1—4.

**Table 1:** Trajectory and speed planning performance benchmark using the RRT* for the obstacle‐free path calculation

	Length (m)		Clearance (m)			Velocity (m/s)			Planned traversal time (s)		$\frac{da_{at}}{dt}\left(m/s^{3}\right)$	
#Traj.	Direct path	Mean ±3σ	Mean of min.	Min. of min.	Mean of mean ±3σ	Mean of mean ±3σ	Mean of max.	Max. of max.	Ours mean	Incr. mean (%)	Mean of max.	Max. of max.
1	30.25	40.84 ± 3.51	3.00	2.24	4.70 ± 0.45	4.83 ± 0.29	9.05	9.68	8.47	4.36	10.98	19.66
2	21.63	24.89 ± 1.64	2.99	2.24	4.07 ± 0.31	4.36 ± 0.34	6.47	7.69	5.72	3.92	8.78	12.17
3	18.71	24.62 ± 2.24	3.04	2.97	4.23 ± 0.33	3.86 ± 0.40	5.76	6.38	6.38	5.62	5.71	9.05
4	25.24	37.85 ± 1.61	3.09	2.85	5.10 ± 0.22	4.50 ± 0.32	8.43	8.97	8.42	5.56	10.52	17.28
5	28.41	59.48 ± 3.05	3.43	2.69	4.91 ± 0.27	5.04 ± 0.38	9.01	10.00	11.81	4.29	12.04	21.07
6	16.14	27.78 ± 48.36	1.68	1.35	2.88 ± 1.50	3.98 ± 1.78	6.15	9.76	6.65	3.84	7.10	17.25
7	14.00	58.74 ± 2.40	2.23	2.00	3.70 ± 0.31	5.48 ± 0.39	9.37	9.85	10.71	2.81	12.29	23.57
8	31.55	52.28 ± 19.49	2.25	2.25	4.08 ± 0.65	5.23 ± 0.86	8.22	9.80	9.98	3.57	10.99	23.55
9	18.17	27.26 ± 6.29	2.50	2.50	4.07 ± 0.42	3.95 ± 0.36	5.96	6.52	6.90	4.09	6.47	9.86

*Note*. RRT: Rapidly exploring Random Tree.

**Table 2:** Trajectory and speed planning performance benchmark using the PRM* for the obstacle‐free path calculation

	Length (m)		Clearance (m)			Velocity (m/s)			Planned traversal time (s)		$\frac{da_{at}}{dt}\,(m/s^{3})$	
#Traj.	Direct path	mean ±3σ	Mean of min.	Min. of min.	Mean of mean ±3σ	Mean of mean ±3σ	Mean of max.	Max. of max.	Ours mean	Incr. mean (%)	Mean of max.	Max. of max.
1	30.25	40.53 ± 3.29	3.02	2.55	4.66 ± 0.47	4.81 ± 0.34	9.11	9.65	8.43	4.43	11.56	24.79
2	21.63	24.76 ± 0.90	2.93	2.30	4.02 ± 0.41	4.31 ± 0.32	6.44	6.89	5.74	4.11	8.82	11.41
3	18.71	24.68 ± 2.68	3.04	2.89	4.26 ± 0.33	3.84 ± 0.46	5.73	6.35	6.43	5.77	5.91	9.82
4	25.24	38.04 ± 1.68	3.09	2.85	5.11 ± 0.21	4.47 ± 0.33	8.53	9.13	8.51	5.57	10.52	16.09
5	28.41	59.33 ± 2.87	3.51	2.95	4.89 ± 0.21	5.03 ± 0.41	8.99	9.74	11.80	4.37	11.62	27.19
6	16.14	19.36 ± 17.88	1.52	1.35	2.65 ± 0.63	3.73 ± 0.70	5.41	8.67	5.17	3.29	5.49	16.37
7	14.00	58.08 ± 3.73	2.21	1.75	3.57 ± 0.42	5.46 ± 0.37	9.33	10.11	10.65	2.95	12.08	22.19
8	31.55	49.70 ± 12.61	2.25	2.00	4.03 ± 0.50	5.15 ± 0.94	7.86	9.35	9.64	3.64	10.59	21.62
9	18.17	27.07 ± 5.78	2.50	2.50	4.08 ± 0.34	3.91 ± 0.44	6.00	6.55	6.92	4.33	6.28	9.55

*Note*. PRM: Probabilistic RoadMap.

**Table 3:** Execution time of the different steps of our trajectory generation approach, using the RRT* obstacle‐free path planner

		Execution times (s)				
				Speed planning		
#Traj.	$A\text{vg.}N_{wp}$	Planning obs.‐free	Path smoothing	Ours	Alg. (Hoffmann et al., 2008)	Overall ±3σ
1	57	1.0086	0.0988	0.1663	0.0006	1.3132 ± 0.1414
2	35	1.0108	0.0447	0.0369	0.0003	1.1305 ± 0.0693
3	35	1.0103	0.0598	0.0352	0.0003	1.1429 ± 0.0624
4	53	1.0103	0.1019	0.1283	0.0004	1.2790 ± 0.1119
5	85	1.0110	0.1846	0.5512	0.0007	1.7862 ± 0.4205
6	39	1.0405	0.0718	0.1207	0.0003	1.2711 ± 0.9011
7	83	1.0105	0.1761	0.5110	0.0007	1.7369 ± 0.3989
8	74	1.0106	0.1503	0.3836	0.0006	1.5836 ± 0.6583
9	38	1.0116	0.0620	0.0487	0.0003	1.1605 ± 0.1085

*Note*. RRT: Rapidly exploring Random Tree.

**Table 4:** Execution time of the different steps of our trajectory generation approach, using the PRM* obstacle‐free path planner

		Execution times (s)				
				Speed planning		
#Traj.	$A\text{vg.}N_{wp}$	Planning obs.‐free	Path smoothing	Ours	Alg. (Hoffmann et al., 2008)	Overall ±3*σ*
1	57	1.0558	0.0928	0.1527	0.0005	1.3371 ± 0.1536
2	35	1.0477	0.0432	0.0366	0.0003	1.1646 ± 0.0854
3	35	1.1189	0.0564	0.0336	0.0003	1.2461 ± 0.1146
4	53	1.0298	0.0997	0.1220	0.0004	1.2869 ± 0.0977
5	83	1.0624	0.1809	0.4936	0.0006	1.7765 ± 0.3199
6	27	1.2131	0.0419	0.0264	0.0002	1.3179 ± 0.3177
7	82	1.0433	0.1515	0.4654	0.0006	1.6982 ± 0.3267
8	71	1.1049	0.1391	0.3063	0.0006	1.5887 ± 0.3288
9	38	1.2492	0.0591	0.0466	0.0003	1.3903 ± 0.2201

*Note*. PRM: Probabilistic RoadMap.

The calculated performance parameters for the trajectories and speed plans are shown in Tables 1 and 2, and for the execution times in Tables 3 and 4. Each row of the tables shows the following performance statistics for the corresponding trajectory query: regarding the path length, the direct distance from initial to target point and the average trajectory length with its corresponding 3σ uncertainty; regarding the path clearance, the mean of the minimum clearance, the minimum overall clearance for this query, and the resulting mean path clearance with its corresponding 3σ uncertainty; regarding the planned velocity, the mean traversal velocity with its corresponding 3σ uncertainty, the mean maximum velocity, and the maximum overall velocity for this query; regarding the traversal time, its mean for each query and the relative increase of the traversal time required by the speed plan smoothing, which effectively compares our speed plan with that resulting from the approach by Hoffmann et al. (2008); and regarding the acceleration derivative, the mean of its maximum value and its overall maximum value for each query are shown. The execution times for each main subpart of our trajectory planning approach are shown in Tables 3 and 4, where the speed plan calculation time for the algorithm by Hoffmann et al. (2008) is also shown, along with the resulting average number of waypoints for each query.

The results, see Tables 1 and 2, show that for queries outside buildings, with enough free space for the path, both obstacle‐free planning algorithms, PRM* and RRT*, provide similar performance and result also in comparable metrics for the speed plan. The biggest differences are to be expected in trajectory queries that require navigation through narrow spaces, such as 6, 7, and 8, see Figure 9, and they effectively occur in Queries 6 and 8. In these two queries, the PRM* algorithm provides on average shorter trajectories with a smaller standard deviation in length, and therefore, resulting also in faster traversal times. In these cases, the clearance of the trajectory is also better with the PRM* as shown by the lower values of its standard deviation. The overall mean velocity of the traversal of the planned trajectories ranges between 4 and 5.5 m/s, with maximum speed values of up to 10 m/s. In all the trajectory queries, we only incur an increased path traversal time of 3–6% when comparing our speed plan smoothing result with the corresponding result from the Hoffmann et al. (2008) algorithm. The only trajectory queries that show low values for clearance are 6, 7, and 8, which require the drone to fly inside an area with a width of 5.25 m, with a maximum achievable clearance in these regions of 2.62 m. Regarding the clearance of Query 9, the initial point of this trajectory has a clearance of 2.5 m. Overall, the planner always provided a feasible trajectory for all the executed queries and, except for the cases explained, the paths showcase a very good clearance and are reasonably distant from obstacles.

Regarding the execution times on the drone’s onboard computer, see Tables 3 and 4, the overall planning takes on average 1.5 s, from which 1.0 s is reserved to the obstacle‐free path planning and the rest is dedicated to the path smoothing and the speed plan smoothing. The execution time of the speed plan smoothing depends on the trajectory length, as it is required to repeatedly solve a linear equations system, the size of which depends on the number of waypoints of the trajectory. For comparison, the shorter trajectory lengths result from Queries 2, 3, 6, and 9 and require only 25–50 ms for the computation of the speed plan. Although the algorithm by Hoffmann et al. (2008) is significantly faster, it results, as discussed in Section 5.2.1, in speed plans that are unfeasible for high velocities. We favor, therefore, the use of our approach, because overall, these execution times are good for our target application in this research study.

### 5.3 Evaluation of the overview obstacle map

In this section, we evaluate quantitatively the quality of the overview obstacle maps obtained using our method, described in Section 3.1, when executed during flight onboard the drone. To acquire these maps, the drone flew autonomously a regular survey flight trajectory. The drone navigates through the survey trajectory by using the algorithms described in Sections 4.1 and 4.2, and images are processed on‐the‐fly to generate the overview obstacle map using the algorithm explained in Section 3.

The regular survey flight trajectory is generated based on two configuration parameters, the desired map generation time and the GPS corners of the area of interest. The number of images that can be acquired is calculated through the approximate processing time per image required by the onboard computer. The image acquisition positions for the creation of the overview map are distributed along a horizontal grid at a constant height and spaced to result in an equal image overlap in both directions of the grid. In this situation, for a given camera (with calibrated focal length and intrinsic parameters), the resulting image overlap depends on the height and the number of images. Therefore, the height of the grid is calculated from the desired image overlap. Additionally, a minimum height of 34 m and a maximum height of 100 m for the survey flight are enforced as a safety measure.

The evaluation is performed on three maps generated in different areas: *scene1* (Fiure 10), *scene2* (Figure 11) and *scene3* (Figure 12). Each of these figures shows an overview image of the area with a red contour around the effectively mapped area, and the corresponding SfM sparse model, the surface model, and the resulting Octomap obstacle map at 1 m resolution. The sparse model consists of the camera positions, shown with camera frustums, and the triangulated points and lines.

**Figure 10:**
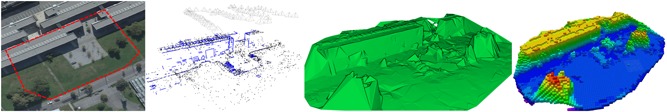
*Scene1*—Obstacle map of size 105 m × 75 m generated onboard in ≈2.75 min from 56 images, from left to right: “Google Earth ©2015” image with red contour around the effectively mapped area, the SfM sparse model, the surface model, and the Octomap obstacle map with 1 m resolution. SfM: Structure from Motion [Color figure can be viewed at wileyonlinelibrary.com]

**Figure 11:**
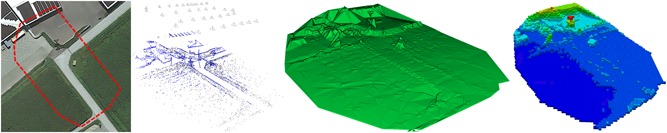
*Scene2*—Obstacle map of size 103 m × 75 m generated onboard in ≈2.75 min from 35 images, from left to right: “Google Earth ©2015” image with red contour around the effectively mapped area, the SfM sparse model, the surface model, and the Octomap obstacle map with 1 m resolution. SfM: Structure from Motion [Color figure can be viewed at wileyonlinelibrary.com]

**Figure 12:**
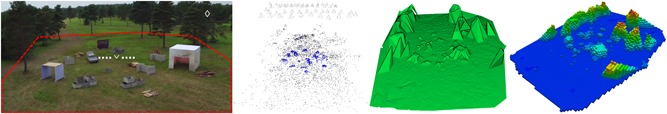
*Scene3*—Obstacle map of size 106 m × 75 m generated onboard in ≈2.75 min from 52 images, from left to right: image with red contour around the effectively mapped area, the SfM sparse model, the surface model, and the Octomap obstacle map with 1 m resolution. SfM: Structure from Motion [Color figure can be viewed at wileyonlinelibrary.com]

The mapping operations took overall onboard and during a flight around 2.75 min. In all, 35–56 grayscale images with a resolution of 1,280 × 720 pixels and 85% vertical and 72% horizontal overlaps were acquired and processed to generate the georeferenced map models, resulting in maps with an approximate size of 105 m × 75 m.

In the context of the qualities of the three reconstructions, the following elements are usually well represented in the map:

- Some example elements are shown zoomed in Figure 13.
  **Figure 13:**
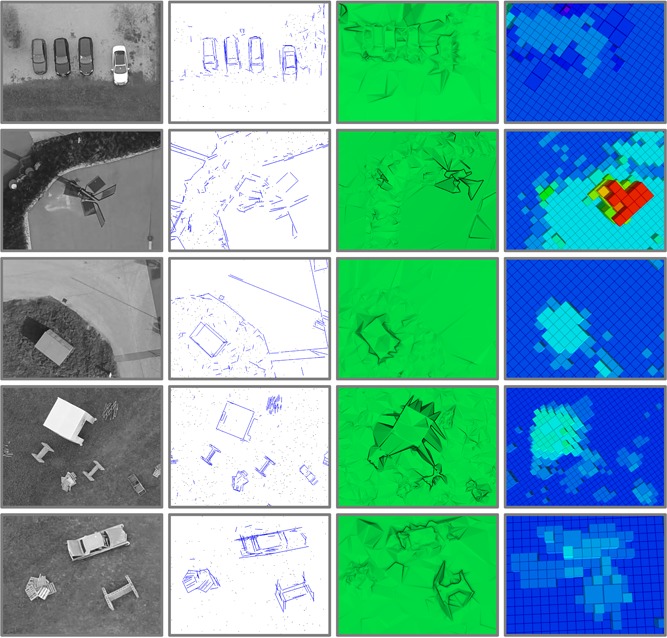
Detail of smaller elements that are well reconstructed and included into the georeferenced overview obstacle map: four cars from *scene1*; a pylon with solar panels and a structure from *scene2*; and the hut, debris, and a car from *scene3* [Color figure can be viewed at wileyonlinelibrary.com]
- *Scene1*—Figure 10: big buildings, cars, man‐made objects with visually distinguishable lines, road edges, and, to a certain extent, grass and part of the trees.
- *Scene2*—Figure 11: a building, man‐made structures with visually distinguishable lines, road edges, and grass.
- *Scene3*—Figure 12: woodland area, human‐made debris, cars, and, to a certain extent, grass, and part of the trees.

**Figure 14:**
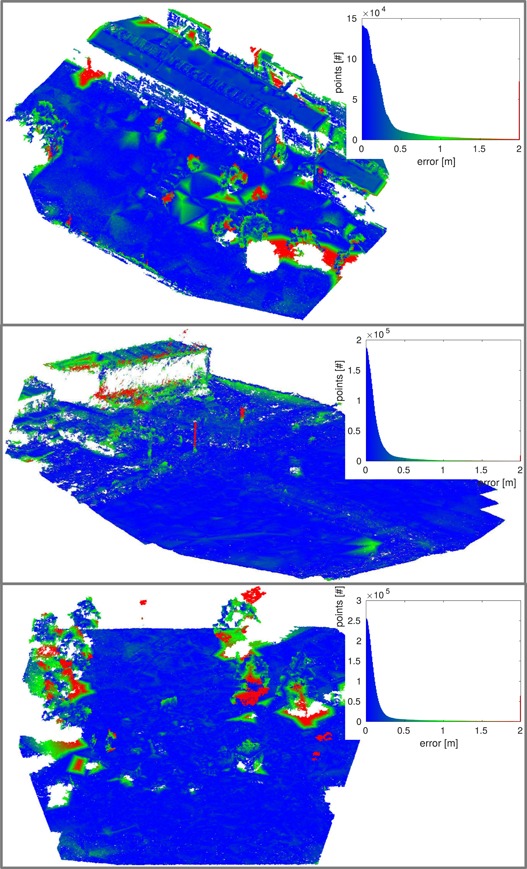
Quantitative accuracy evaluation of our mapping method against dense point‐clouds obtained using *Pix4D*. The top image corresponds to *scene1*, the middle to *scene2*, and the bottom to *scene3*. The reconstruction error is calculated as the distance between each dense point and the onboard calculated mesh model. The dense point‐clouds are color‐coded according to the reconstruction error, where the color‐to‐distance correspondence** is shown in the histograms. The reconstruction error distribution is shown graphically by the histograms in the figure and numerically in Table 5 [Color figure can be viewed at wileyonlinelibrary.com]

We assess the accuracy of our onboard mesh models against a dense point‐cloud reconstruction that was obtained using the photogrammetry software *Pix4D* 6. To perform the accuracy assessment calculation, the point‐cloud was first registered to the mesh model using the Iterative Closes Point (ICP) algorithm, second cropped along the borders, because the area of interest is in the middle of the model, and third the distance of each dense point to the mesh was calculated, which is used as estimate of the reconstruction error. In this calculation, the error was saturated to 2 m so that its distribution, shown in the histograms, can be better appreciated. The registration of the dense point‐cloud to the mesh model is performed to extract the slight differences in the georeferenciation results, which would otherwise affect our accuracy assessment. The reconstruction error between these high‐quality point‐clouds and their corresponding onboard calculated mesh models is shown graphically in Figure 14 and numerically in Table 5. The number** of dense points that for each model have an error of 0.5 m or less are: 85.2% for *scene1*, 93.2% for *scene2*, and 88.7% for *scene3*.

**Table 5:** Quantitative accuracy evaluation of our mapping method

	Percentage (%) of points with a reconstruction error ≤d (m)							
d (m)	0.25	0.50	0.75	1.00	1.25	1.50	1.75	2.00
*scene1*	69.49	85.21	90.59	93.69	95.73	97.05	97.97	98.55
*scene2*	84.60	93.19	96.46	97.98	98.76	99.30	99.59	99.73
*scene3*	82.31	88.68	91.83	94.11	95.78	96.91	97.80	98.52

*Note*. This data corresponds to the histograms from Figure 14. The reconstruction error is calculated as the distance between the dense points from the *Pix4D* reconstructions, displayed in Figure 14, to the onboard calculated meshes, see Figures 10–12.

From the accuracy evaluation, we can assess that there are some areas, which tend to be not well mapped by our onboard overview mapping solution, due to the fact that our mesh is derived from a sparse 3D model. Some examples of challenging regions of the scene are:

- Textureless surfaces or with too fine a texture for the current image resolution and acquisition height, causing problems for the point‐feature matching. For example, asphalt at 34 m with our drone’s camera is problematic.
- Vegetation, branches, and foliage. For example, in particular trees.
- Dark and untextured areas, for example, asphalt or façades in shadows, are not reconstructed properly when using point‐based SfM. The subsequent Delaunay triangulation (Labatut et al., 2007), therefore, tends to close these nonreconstructed areas with big triangles, for example, see the building near the border in *scene2*.

For the purpose of navigation, our current solution to this issue, discussed in Section 3.2, is to update the Octomap obstacle map on‐the‐fly by fusing the depth maps from the stereo‐heads.

### 5.4 Evaluation of autonomous navigation

**Figure 15:**
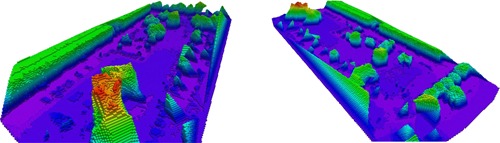
Overview obstacle map environment used by the trajectory planner in our autonomous navigation experimental flight. The map of size 55 m × 170 m × 40 m was generated from 195 images with a higher than onboard resolution of 4,912 × 3,264 pixels using our method, see Section 3.1, with the same parameter configuration used for onboard real‐time execution. Displayed voxel‐grid resolution: 0.50 m [Color figure can be viewed at wileyonlinelibrary.com]

For the experimental evaluation of our navigation architecture, described in Sections 4.1 and 4.2, the drone is set up to perform autonomous obstacle‐free navigation on an area, for which we have previously acquired an obstacle map. For this purpose, we acquired the obstacle map of the test area of size 55 m × 170 m × 40 m, shown in Figure 15.

The Octomap resolution used by the planner on this map is 1 m. A long trajectory on this area can easily reach a length of 100 m, allowing us to test the capabilities of our navigation architecture. The trajectory planner was set to use the PRM* algorithm for the generation of raw obstacle‐free paths, and it was configured with a maximum speed and acceleration of 4.0 m/s and $0.15g=1.47{\text{m/s}}^{2}$, respectively. Otherwise, the planner is configured as described in Section 5.2.

We utilize the drone equipped as explained in Section 5.1. All the algorithms are run onboard the drone, except for a user interface that runs on a laptop that allows a user to teleoperate the drone by using what we term a point‐and‐click interface. By clicking on a point of the obstacle map, the drone is commanded to navigate to a waypoint of 1.5 m over the clicked point. Therefore, the user is able to teleoperate the drone from a laptop, connected through WiFi, and issue the following commands: takeoff, point‐and‐click navigation, stop, and land. The onboard computer is configured appropriately, so that all the onboard ROS modules are able to continue to intercommunicate in the case of an eventual WiFi disconnection.

**Figure 16:**
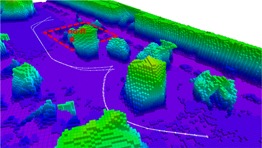
Trajectories flown by the drone in the experimental evaluation of our autonomous navigation architecture [Color figure can be viewed at wileyonlinelibrary.com]

During the flight, the drone was first commanded to take off. Then, the point‐and‐click interface was used to designate the next waypoint, so that the drone planned an obstacle‐free trajectory to it, which was immediately executed autonomously. After reaching the end of the trajectory, the user then issued, by clicking on the user interface, the next waypoint. The drone was able to navigate in this manner to all four commanded waypoints by following the trajectories shown in Figures 16 20. The information in these figures is displayed following the same convention as for Figures 6 and 7. A video of the experiment is available online^7^.

**Figure 17:**
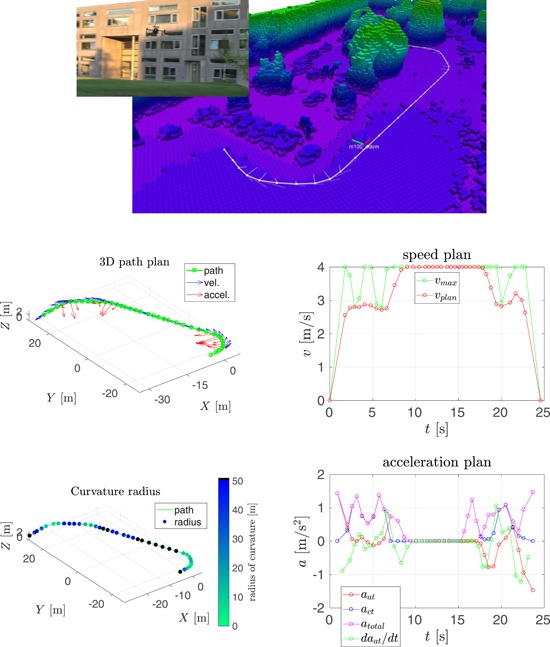
Experimental flight trajectory 1—(top) Visualization of the planned trajectory, (middle‐left) trajectory path in 3D, (bottom‐left) path color‐coded with the radius of curvature, (middle‐right) speed, and (bottom‐right) acceleration plans [Color figure can be viewed at wileyonlinelibrary.com]

**Figure 18:**
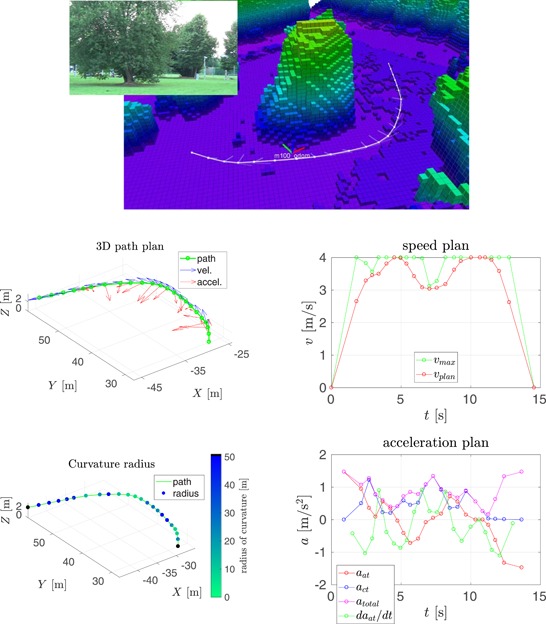
Experimental flight trajectory 2—(top) Visualization of the planned trajectory, (middle‐left) trajectory path in 3D, (bottom‐left) path color‐coded with the radius of curvature, (middle‐right) speed, and (bottom‐right) acceleration plans [Color figure can be viewed at wileyonlinelibrary.com]

**Figure 19:**
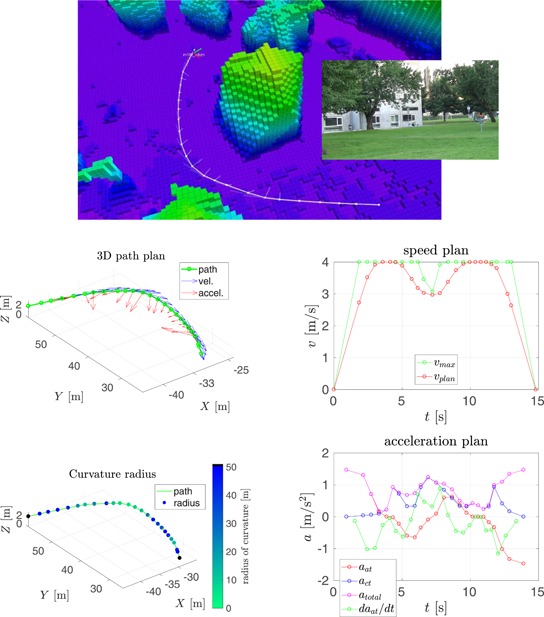
Experimental flight trajectory 3—(top) Visualization of the planned trajectory, (middle‐left) trajectory path in 3D, (bottom‐left) path color‐coded with the radius of curvature, (middle‐right) speed, and (bottom‐right) acceleration plans [Color figure can be viewed at wileyonlinelibrary.com]

**Figure 20:**
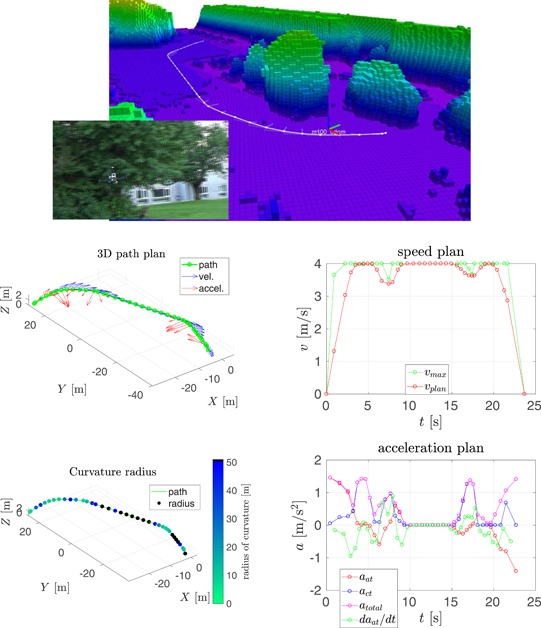
Experimental flight trajectory 4—(top) Visualization of the planned trajectory, (middle‐left) trajectory path in 3D, (bottom‐left) path color‐coded with the radius of curvature, (middle‐right) speed, and (bottom‐right) acceleration plans [Color figure can be viewed at wileyonlinelibrary.com]

A side benefit of georeferencing the obstacle maps is that we are able to perform geofencing and to set no‐fly areas specifying only their GPS corner points, so that the trajectory planner regards them as obstacles. This feature was evaluated by marking a parking area as a no‐fly zone, see Figure 16.

Our navigation results are summarized by the performance parameters shown in Table 6. The drone navigated safely in the mapped area reaching maximum speeds of 4.0 m/s and navigated at an average speed of 2.98 m/s while traversing trajectories with an average length of 61.6 m.

**Table 6:** Trajectory planning and control performance in our autonomous navigation experiment

		Execution time (s)		Speed (m/s)	
#Traj.	Path length (m)	UI + plan	Navigation	Avg.	Max.
1	77.24	2.52	26.6/25.5	2.90	4.00
2	43.53	1.66	15.7/14.9	2.77	4.00
3	44.96	2.09	15.6/14.9	2.88	4.00
4	80.52	2.88	24.7/23.8	3.26	4.00
Avg.	61.6	2.29	20.6/19.8	2.98	4.00

*Note*. All the values shown in the table are derived from a data log of the flight shown in Figures 16–20, considering the times at which ROS messages were published. The UI + plan time corresponds to the whole trajectory generation onboard plus the delay interval between clicking on the next target point on the User Interface (ROS Rviz) and receiving the goal point in the drone through WiFi. The traversal time denotes the [(real)/(planned)] time it took the controller to perform the commanded trajectory, where the left column shows the (real) traversal time and the right column the expected or (planned) traversal time. The last two columns show the average and maximum navigation speeds during each trajectory. The last row reports the average value for each column.

ROS: Robot Operating System; UI: user interface.

This experiment demonstrates the capability of our trajectory planner to generate trajectories to navigate safely utilizing maps obtained by means of our onboard real‐time capable photogrammetry method. The planner provided feasible speed, acceleration, and time‐of‐passage plans constrained by maximum speed and acceleration configuration parameters. The drone successfully performed obstacle‐free navigation and respected the velocity and acceleration constraints set in the configuration of the trajectory planner.

## 6 CONCLUSIONS

In this study, we proposed a vision‐based method for a drone to generate onboard on‐the‐fly an overview obstacle map that is immediately available for navigation tasks. Flying at the overview altitude the drone is able on its own to map a GPS‐defined region of interest in a short period of time. In this process, the reconstruction of man‐made objects and infrastructure is enhanced by exploiting 3D lines. The actual size and level of detail of the acquired map depends on the utilized camera, lens, and flight altitude. Based on the constrained onboard computational power of our drone, a map of size 105 m × 75 m is acquired in ≈2.75min. In the experiments, we quantitatively evaluated the accuracy of the acquired maps. We demonstrated the usability of the generated obstacle map in an autonomous flight experiment. The georeferenciation of the map is performed using only GPS measurements, which offers a general solution for search and rescue scenarios. The performed georeferenciation provides an absolute accuracy up to the positioning precision of the onboard GPS sensor.

Our experiments demonstrate the capabilities for an autonomous drone to acquire the obstacle map of a moderately sized area using a vision‐based method and, by using the acquired map, to immediately perform near‐ground obstacle‐free navigation in that area. The overview obstacle map is up‐to‐date and through it we can generate paths away from obstacles. In contrast to pure reactive obstacle avoidance or to trajectory planning with an outdated potentially invalid map, the up‐to‐date overview map should enable an overall increased mission execution efficiency. The reason for this is that the availability of the overview map during an autonomous mission allows the robot to focus on direct task objectives rather than on exploration. Although onboard mapping approaches will continue to be improved, our proposed vision‐based approach represents a step forward in the fast deployment of autonomous drones in unknown outdoor environments.

## 7 FUTURE WORK

There are still regions of the scene, which are challenging to reconstruct using point‐based SfM. A possible approach to deal with them is to densify the point‐cloud, for example, by using patch‐based multi‐view stereo (PMVS) (Furukawa & Ponce, 2010), SURE (Rothermel, Wenzel, Fritsch, & Haala, 2012), PlaneSweepLib (PSL; (Häne, Heng, Lee, Sizov, & Pollefeys 2014), or the work by Shekhovtsov, Reinbacher, Graber, and Pock (2016). Often times, the usage of these algorithms requires more computation power than available on current onboard computers of drones, which motivates the development of faster algorithms for the map densification. Another approach is the utilization of view‐planning methods (Roberts et al., 2017; Schmid et al., 2012), for instance, the approach (Mostegel, Rumpler, Fraundorfer, & Bischof, 2016) results in a set of images adapted to the challenging elements present in the scene, for example, by providing short baseline images for vegetation.

In our approach, the drone is localized in the overview obstacle map by using GPS + IMU fusion. This poses a problem for collision avoidance because a typical GPS + IMU state estimate can drift a few meters, particularly for longer flight times and during navigation close to the ground and among buildings. The localization precision against the overview map can be further improved by additionally fusing vision‐based localization methods. However, feature‐based matching between images acquired at a large and a close distance is challenging and thus provides options for future research. An enhanced relocalization capability would also provide improvements for collaborative mapping, map reuse, and map sharing between different robots and devices.

Next to our method, maps are also generated using SLAM approaches that combine a VO or VIO front‐end with a bundle adjustment back‐end (e.g., Forster, Lynen, Kneip, & Scaramuzza, 2013, 2017; Schmuck & Chli, 2017; T. Schneider et al., 2018). In the context of overview obstacle maps, a detailed benchmark comparison of the mapping accuracy and processing time requirements of our SfM‐based method against those algorithms would be of interest.

The experimental focus on this study was performed to support the main contribution in the mapping task. The further benchmarking of the trajectory planner in experiments and simulation, for instance, based on the benchmarks (Mettler, Kong, Goerzen, & Whalley, 2010; Nous, Meertens, Wagter, & de Croon, 2016), is left as future work.

## ACKNOWLEDGMENTS

This study has been supported by the Austrian Science Fund (FWF) project V‐MAV (I‐1537), the Austrian Research Promotion Agency (FFG) project FreeLine (Bridge1/843450), and OMICRON electronics GmbH. The authors would like to thank the hardware donation from DJI, granted as a part of the *Graz Griffins* team participation in the *2016 DJI Developer Challenge*.

## Footnotes

1
https://developer.dji.com/challenge2016/

2
http://icg.tugraz.at/—Institute of Computer Graphics and Vision, Graz University of Technology.

3
https://github.com/OctoMap/octomap

4
http://wiki.ros.org/octomap/_mapping

5 The code is available inside the Octomap source‐code repository.

6
https://pix4d.com/

7 Video of the experimental flight: https://youtu.be/9DXzIGKKqbU.
